# ﻿Broadly sympatric occurrence of two thief ant species *Solenopsisfugax* (Latreille, 1798) and *S.juliae* (Arakelian, 1991) in the East European Pontic-Caspian region (Hymenoptera, Formicidae) is disclosed

**DOI:** 10.3897/zookeys.1187.105866

**Published:** 2023-12-21

**Authors:** Sándor Csősz, Bernhard Seifert, Márk László, Zalimkhan M. Yusupov, Gábor Herczeg

**Affiliations:** 1 HUN-REN-ELTE-MTM Integrative Ecology Research Group, Pázmány Péter ave 1/C, Budapest 1117, Hungary ELTE-Eötvös Loránd University Budapest Hungary; 2 Department of Systematic Zoology and Ecology, Institute of Biology, ELTE-Eötvös Loránd University, Pázmány Péter ave 1/C, Budapest 1117, Hungary HUN-REN-ELTE-MTM Integrative Ecology Research Group Budapest Hungary; 3 Department of Entomology, Senckenberg Museum of Natural History Görlitz, Am Museum 1, 02826 Görlitz, Germany Senckenberg Museum of Natural History Görlitz Görlitz Germany; 4 Tembotov Institute of Ecology of Mountain Territories of RAS, Nalchik, 360051, Russia Tembotov Institute of Ecology of Mountain Territories of RAS Nalchik Russia

**Keywords:** Biogeography, cryptic species, dimorphism, exploratory analyses, morphometry, species delimitation

## Abstract

This paper presents numeric morphology-based evidence on the broadly overlapping distribution of two thief ant species *Solenopsisfugax* (Latreille, 1798) and *S.juliae* (Arakelian, 1991) in the East European Pontic-Caspian region. The paper integrates two autonomous data collections and independent analyses performed by different researchers, using different equipment, considering different character combinations, and evaluating partially different samples. Five type series, the neotype series of *Solenopsisfugax* (Latreille 1798) and the type series of *S.flavidula* (Nylander, 1849), S. (Diplorhoptrum) fugax var. furtiva Santschi, 1934, S. (Diplorhoptrum) fugax var. pontica Santschi, 1934, S. (Diplorhoptrum) fugax var. scytica Santschi, 1934 were nested in one cluster and we propose the junior synonymy of the latter four taxa names with *S.fugax*. The other cluster contained only one type specimen of *Solenopsisnitida* (Dlussky & Radchenko, 1994) measured from AntWeb images. The naming of this cluster was based on both verbal statements and measurements of gynes given in the original description of *Solenopsisjuliae* (Arakelian, 1991), which represents the oldest available name for this cluster. Hence, *S.nitida* is proposed as junior synonym of *S.juliae*. *Solenopsiscypridis* Santschi, 1934 is raised to species rank based on investigation of worker and gyne type specimens.

## ﻿Introduction

The Myrmicine ant genus *Solenopsis* Westwood 1840 is distributed worldwide over the tropical, subtropical, and temperate zones. Approximately 200 species are currently recognized, with 80% of these being distributed in the Neotropical and South Nearctic zone. The catalog of Bolton (2023) lists 24 species plus four subspecies for the West Palaearctic region, mainly described from the African and European parts of the West Mediterranean and temperate France. The Palaearctic species are essential elements of myrmecocenoses in xerothermous grassland and forest-steppe ecosystems. In oligotrophic xerothermous grasslands of Central Europe, the so-called thief ant *Solenopsisfugax* (Latreille, 1798) may build up maximum densities of 92 nests /100 m^2^ (Seifert 2017) or may constitute up to 20–25% of total ant biomass (Wagner and Wieser 2021). This subterranean species is a true cleptobiont in probably all larger ant species occurring in its habitats. However, it does not depend on this lifestyle as it is necrophagic, strongly trophobiotic, and preys on diverse developmental stages of soil invertebrates. Colonies with strongly cleptobiotic nutrition are apparently selected for uniform smallness of workers, whereas those using diverse food sources usually contain both minor and major workers (Hölldobler 1965). Other researchers have also documented this size dimorphism (e.g., Galkowski et al. 2010; pers. obs.).

The taxonomy of the genus became highly complicated in the West Mediterranean area due to 15 taxa described from France and Corsica. After Latreille (1798) had described *S.fugax*, Santschi (1934) added three taxa, whereas Bernard (1950, 1959, 1978) described as many as 15 taxa, among these 14 from France and four of them from a small area near Banyuls sur Mer. There is little doubt that this inflated list contains many synonyms. Galkowski et al. (2010) attempted to bring some order into this confusing heritage. Fixing a neotype, they redescribed *Solenopsisfugax* and delimited four species groups: the *S.fugax* group, the *S.debilior* group, the *S.lusitanica* group, and the *S.orbula* group. The authors commented, “A largest and most extensive collect will be necessary for a new morphological and molecular approach of the genus and to go further in understanding the Solenopsis of France” (Galkowski et al. 2010: 151). We agree, but are unable to give data-based, thoughtful comments on the situation in the Palaearctic west of 8°E; it may be that these species groups represent only a little more than four species. In contrast, the situation farther east is easier to conceive. Therefore, we take the occasion to progress for at least one segment of *Solenopsis* taxonomy for the area of Central Europe, the Balkans, Ukraine, Asia Minor, and the Caucasian region. After Seifert (2018) stated that only a single species occurs north of the Alps, which is by type comparison *Solenopsisfugax*, we became recently aware using morphometric examination that two distinct morphospecies occur in this eastern region in sympatry. One of them is *Solenopsisfugax*, and we provide an argument that the eastern species should be *Solenopsisjuliae* (Arakelian 1991). We therefore consider all relevant taxa described from this eastern region or near to it to establish these names.

Morphometric investigation of *Solenopsis* is challenging and requires high-resolution stereo microscopy as the worker thief ants often have a minute size of less than 2 mm in total length. The size dimorphism of workers adds a second complication (e.g., Hölldobler 1965; Galkowski et al. 2010). Gyne dimorphism is also not unusual in *Solenopsis*, confirmed in both *S.geminata* (Fabricius, 1804) and *invicta* Buren, 1972 (McInnes and Tschinkel 1995; Helms and Godfrey 2016), and it also appeared in the two species considered here with the result that microgynes of *S.fugax* have a similar absolute size as do macrogynes of *S.juliae*. This size dimorphism requires corrections for the strong allometric effects to show which morphological differences are “species specific” and to improve the performance of principal component analyses. We follow here the working rationale of species hypothesis formation by different forms of exploratory data analyses of continuous morphometric data and checking these hypotheses by linear discriminant analysis (Seifert et al. 2014; Csősz and Fisher 2016).

We repeat the parallel data collection procedures of previous papers (Csősz and Seifert 2003; Seifert and Csősz 2015). Both authors had spent a substantial amount of time investigating the *Solenopsisfugax* problem before they realized they were working on the same issue. This led to the recording of different, but partially identical character sets and samples. It is important to note that only 35% of the total 62 worker samples were measured by both SC and BS. We decided to continue with the different approaches, which each followed a robust statistical procedure. Three factors should be noted in this context: (i) this two-dataset setup provides an excellent ground to test whether or not the differently collected morphometric data bias our findings, (ii) a post-hoc synchronization of these different systems was avoided as this would have been overly time-consuming, and (iii) a data synchronization was not performed as this would have implied a loss of information. The disadvantage of the complex presentation is compensated by the pleasant fact that independent approaches led to the same conclusion in the differentiation of cryptic species. Finally, the importance of our research is beyond simply understanding another fragment of European biodiversity; our study also shows that morphometry considering allometry-driven polymorphism is superior in performance compared to subjective evaluation of discrete traits or to presenting a few anecdotal morphometric measurements.

## ﻿Materials and methods

### ﻿Material examined

Altogether 63 worker nest samples were investigated, 42 nest series by Seifert (hereafter BS), 52 by Csősz (SC), and 35% of the total were investigated by both observers. In addition, BS measured 26 gyne samples with 41 specimens. We borrowed and investigated all type series of species described from the target region: *S.fugax* neotype series, *S.flavidula* Nylander 1849, *S.fugaxfurtiva* Santschi, 1934, *S.fugaxpontica* Santschi, 1934, *S.fugaxscythica* Santschi, 1934, and *S.fugaxcypridis* Santschi, 1934. We had to rely upon the morphometric data from images stored in the online virtual collection Antweb.org (AntWeb 2023) in the case of types stored in Kyiv and Moscow due to the Russo-Ukrainian war. Even though we have measured high-resolution AntWeb images with the uttermost caution, we carefully concluded that those image-based morphometric data are in accordance with the latest results on applicability of the virtual collection data (Csősz et al. 2023). The full list of examined material is listed in Suppl. material 1 including locality information, number of measured individuals, and the depositories.

### ﻿Protocol for morphometric character recording

**Figure 1.**
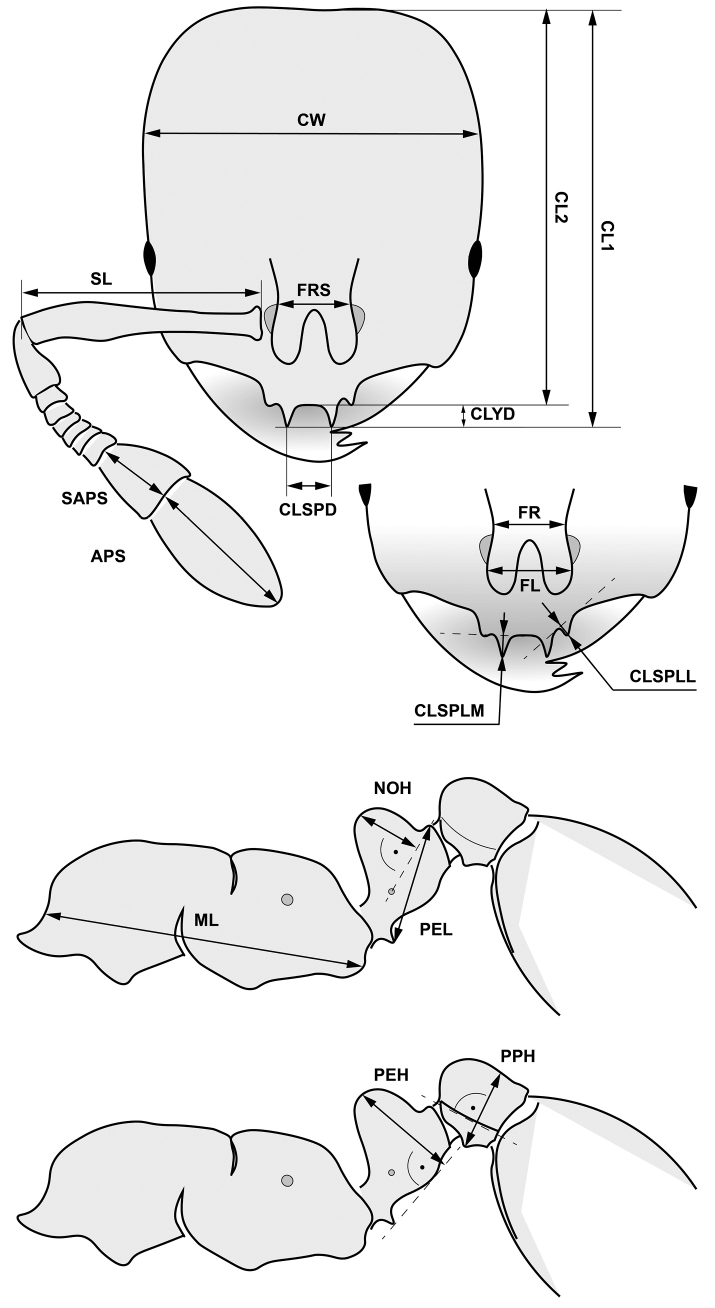
Definition of morphological characters of the *Solenopsis* workers measured in this study. Head in dorsal view with measurement lines for CL1, CL2, CW, FRS, ClSpD, ApS, SApS, and SL; frontal region of the head dorsum with measurement lines for FR, FL, ClSpLL, and ClSpLM; dorsal view of mesosoma with measurement lines for ML, PEH, PEL, NOH, and PPH (for definitions, see Table 1).

**Figure 2.**
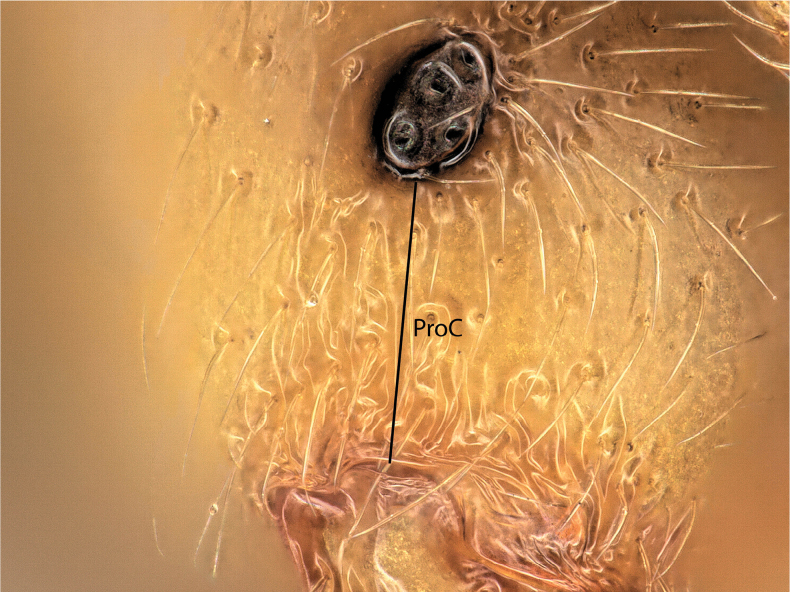
Definition of preocular distance (PROC) of the *Solenopsis* workers measured in this study (for details, see Table 1).

All measurements were made with a cross-scale graticule at µm precision using a pin-holding stage, permitting rotations around X, Y, and Z axes with an Olympus SZX16 stereomicroscope with a 1.6× Plan Apochromat objective at a magnification of ×240 for each character (SC); stereomicroscopic and photographic equipment, measurement procedures of BS are as reported in Seifert (2023). Morphometric data are given in µm throughout the paper. Definitions of morphometric characters are listed in Table 1 and are illustrated in Figs 1, 2. Raw data in µm is given in Suppl. materials 1–3.

**Table 1.** Abbreviations for morphometric characters, character definitions and the relevant observers’ initials are presented in different columns. SC = Sándor Csősz, BS = Bernhard Seifert. In case the protocol was identical between the observers, initials of both authors are given.

Abbreviation	Character definition	Observer
CL1	Maximum cephalic length from the median point of a reference line connecting the tips of the large clypeal teeth to the hind margin of head; the head must be carefully tilted to the position with the true maximum.	SC
CL2	Maximum cephalic length from anteromedian margin of clypeus to posteromedian margin of head; the head must be carefully tilted to the position with the true maximum.	BS
CLSPD	Distance of tips of the large paramedian clypeal dents	SC, BS
CLSPLM	mean length of the large paramedian clypeal dents measured from bottom of menisci left and right of the spines	BS
CLSPLL	mean length of the more lateral, smaller clypeal dents measured from bottom of menisci left and right of the spines	BS
CW	maximum width of head capsule posterior of the eyes	SC, BS
CS	The arithmetic means of CL2 and CW as less variable indicator of absolute size	BS
EL	longest eye diameter	BS
FL	Maximum distance of frontal carinae; if no maximum is defined by a constriction, set FR equal to FRS	BS
FR	Minimum distance of frontal carinae; if no minimum is defined by a constriction, set FR equal to FRS	BS
FRS	distance of the frontal carinae immediately caudal of the posterior intersection points between frontal carinae and the lamellae dorsal of the torulus. If these dorsal lamellae do not laterally surpass the frontal carinae, the deepest point of scape corner pits may be taken as reference line. These pits take up the inner corner of scape base when the scape is fully switched caudad and produce a dark triangular shadow in the lateral frontal lobes immediately posterior of the dorsal lamellae of scape joint capsule	SC, BS
FULL FACE VIEW	Dorsal aspect of head with both maximum head width and maximum median head length in visual plane	SC, BS
ML	Mesosoma length; anterior measuring point in workers: transition point of the anterior pronotal slope to the anterior pronotal shield; anterior measuring point in gynes: frontalmost point of the pronotal slope; posterior measuring point in both workers and gynes: caudalmost margin of the propodeal lobe.	SC, BS
MPGR	depth of metanotal groove measured down from tangent of mesonotopropodeal profile	BS
MW	mesosoma width; this is in workers maximum pronotal width, in gynes the maximum mesosoma width frontal of the tegulae	SC, BS
NOH	Maximum height of the petiolar node, measured from the uppermost point of the petiolar node perpendicular to a reference line set from the petiolar spiracle to the dorso-caudal corner of caudal cylinder of the petiole	SC
PEH	Petiole height. A straight imagination of ventral petiolar profile at node level is the reference line perpendicular to which the maximum height of petiole node is measured at node level. This is the height of a section line but not height above all.	SC, BS
PEW	maximum width of petiole	SC, BS
PPW	maximum width of postpetiole	SC, BS
PPH	maximum postpetiole height; the lateral suture of dorsal and ventral sclerites is the reference line perpendicular to which the maximum height is measured.	BS
PROC	preocular distance; the shortest distance between the anterior eye margin and the sharp frontal margin of the gena. Caution: do not confuse this with the beaded rim of the mandible that is often very closely appressed to the genal margin.	BS
SL	maximum straight line scape length excluding the articular condyle as arithmetic mean of both scapes	SC, BS
APS	With the swiveling plane of antennal funiculus in visual plane (defined by the swiveling plane of the hinge joint of pedicellus with scape), maximum median length of apical funiculus segment.	SC
SAPS	With the swiveling plane of antennal funiculus in visual plane, maximum median length of sub-apical funiculus segment.	SC

### ﻿Data preparation

Allometry is the disproportionate change of body shape and other phenotypic traits with growing body size (Lleonart et al. 2000). As body size is in ants strongly dependent on particular environmental conditions during ontogenesis, in particular from larval nutrition (Tschinkel et al. 2003; Molet et al. 2017), removal of allometric variance (RAV) can be considered as approximation of the data to the genetically determined character space. As result, RAV can expose in comparative tables “true” genetically determined interspecific differences and unmask pseudo-differences (Seifert 2008). Furthermore, RAV may increase the performance of some exploratory data analyses – in particular of principle component analyses (PCA; Seifert 2021). If absolute measurements (raw data) are used in a PCA, the first principal component often indicates the size component which is not of interest in separation of cryptic species, and it may blur the analysis. RAV was performed here with two different methods.

To remove effects of the disproportionate size dependent trait size changes, SC calculated within-nest sample regression analyses of absolute measurements with cephalic length CL1 (Cephalic Length, see Table 1) as the independent variable. Head size is one of the most popularly used size indicator in ants (see e.g., Tschinkel et al. 2003; Seifert 2008; Molet et al. 2017). Coefficients applied to calculate residuals are given in Table 2. The resulting slope and intercept of the correction functions were then calculated as arithmetic means from the 52 samples’ slope and intercept values. RAV was then performed using residuals and with these data an alternative prior species hypothesis was generated via PCA. This procedure does not need a previous formation of species hypotheses but accepts unequal contribution of species to the RAV function finally calculated.

**Table 2.** Removal of allometry via regression model (SC). Residuals for nest samples are calculated via regression analyses for every trait on Cephalic Length (CL1) as the independent variable, coefficients x, and intercept to calculate residuals are provided.

	x(CL1)	(Intercept)
CLYD	0.038	1.555
APS	0.239	74.015
SAPS	0.143	5.354
CWB	1.020	-110.076
FRS	0.219	-10.728
CLYW	0.168	-33.917
SL	0.611	-3.445
MW	0.562	-14.713
PEW	0.321	-14.704
PPW	0.296	5.081
ML	1.183	-60.970
PEL	0.400	-11.529
PEH	0.294	26.156
NOH	0.196	2.364

BS performed RAV by following the basic procedure described in Seifert (2008), which uses regression of index values against cephalic size CS as independent variable. The procedure requires a pre-established hypothesis on species identities and results in equal contribution of the considered species to the final RAV function. Slope and intercept were here calculated as the arithmetic mean of the species-specific functions, in this paper of 92 *S.fugax* and 66 *S.juliae* workers. RAV was then performed by calculating the quotient between the real value and the RAV-function value. In order to have a translucent presentation in comparative tables and descriptions, the quotients were then transformed to index values that would be achieved if all worker individuals had the same cephalic size of CS = 480 µm (Table 1). The procedure resulted in a reduction of within-species variance to 29% in CL2/CW, 73% in SL/CS, 76% in CLSPD/CS, and 65% in PEH/CS, but did not show notable effects in the remaining characters. Parameters for RAV in gynes were calculated alone on the basis of data in *S.fugax* as it was the only species with sufficient sample size.

The RAV functions for workers and gynes (sensu BS) are as follows:

CL2/CW_480_=CL2/CW /(-0.5634*CS+1.4390)*1.1685)

SL/CS_480_=SL/(-0.1460*CS+0.7688)*0.6987

FL/CS_480_=FL/(0.0036*CS+0.2223)*0.2241

FR/CS_480_=FR/(0.0131*CS+0.2083)*0.2146

EL/CS_480_=EL/(0.0772*CS+0.0575)*0.0945

PrOc/CS_480_=PROC/(-0.0135*CS+0.1939)*0.1874

CLSPLM/CS_480_=CLSPLM/(-0.0388*CS+0.0750)*0.0564

CLSPLL/CS_480_=CLSPLL/(-0.0239*CS+0.0314)*0.0199

CLSPD/CS_480_=CLSPD/(0.1082*CS+0.0833)*0.1353

ML/CS_480_=ML/(-0.0094*CS+1.1853)*1.1808

MW/CS_480_=MW/(-0.0881*CS+0.6335)*0.5912

MpGr/CS_480_=MPGR/(0.0051*CS+0.0244)*0.0269

PEW/CS_480_=Pew/(0.0259*CS+0.2943)*0.3068

PPW/CS_480_=PPw/(-0.0592*CS+0.3556)*0.3271

PEH/CS_480_=PEH/(-0.1487*CS+0.4487)*0.3773

PPH/CS_480_=PPH/(-0.0011*CS+0.3058)*0.3006

The RAV functions in gynes are:

CL2/CW_850_=CL2/CW/(-0.2881*CS+1.1973)*0.9524

SL/CS_850_=SL/(-0.2677*CS+0.8775)*0.6500

FL/CS_850_=FL/(-0.1515*CS+0.3890)*0.2602

FR/CS_850_=FR/(-0.1466*CS+0.3834)*0.2588

EL/CS_850_=EL/(-0.1448*CS+0.4169)*0.2938

PrOc/CS_850_=PROC/(0.0899*CS+0.0388)*0.1152

CLSPLM/CS_850_=CLSPLM/(0.0108*CS+0.0403)*0.0494

CLSPLL/CS_850_=CLSPLL/(0.0504*CS-0.0304)*0.0125

CLSPD/CS_850_=CLSPD/(0.0011*CS+0.1395)*0.1405

ML/CS_850_=ML/(0.5567*CS+1.5727)*2.0459

MW/CS_850_=MW/(0.7122*CS+0.4779)*1.0833

MH/CS_850_=MH/(0.6220*CS+0.7668)*1.2854

PEW/CS_850_=Pew/(-0.1511*CS+0.5986)*0.4701

PPW/CS_850_=PPw/(-0.2844*CS+0.7683)*0.5265

PEH/CS_850_=PEH/(0.1010*CS+0.4025)*0.4883

PPH/CS_850_=PPH/(-0.1000*CS+0.5682)*0.4832

### ﻿Statistical framework on morphometric data – hypothesis formation and testing

We use the toolkit of exploratory data analysis of continuous morphometric data (Seifert et al. 2014; Csősz and Fisher 2016) followed by confirmatory data analysis.

### ﻿Exploratory analyses via NC-PART clustering

The prior species hypothesis was generated based on workers via Nest Centroid clustering (NC clustering; Seifert et al. 2014) in combination with partitioning algorithms PART (Nilsen and Lingjaerde 2013; Csősz and Fisher 2016) for estimating the number of biologically meaningful clusters. The protocol of this combination was published by Csősz and Fisher (2016), which is now applied with the following specific setups: bootstrap iterations in PART were set to ‘b=1000’, and the minimum size of clusters were set to ‘minSize=5’ for both ‘hclust’ and ‘kmeans’. Data input for this analysis was performed as absolute measurements (raw data) in the analysis of SC and as allometrically corrected index data (plus CS as absolute size indicator) in the analysis of BS. The script was written in R and can be found in Suppl. material 4.

### ﻿Exploratory analyses via PCA using allometrically corrected data

An alternative prior species hypothesis has been generated by SC via the ordinating Principal Component Analysis (PCA) that displays plots in a graphic. Allometries are calculated via regression analyses for every trait on CL1 as the independent variable (see Table 1), and residuals were applied. Coefficients to calculate residuals are provided in Table 2. Shape ratios corrected according to the RAV system of BS were used to demonstrate heterospecificity in a PCA of workers.

### ﻿Confirmatory data analyses (SC)

The validity of the prior species hypothesis imposed by the exploratory processes was tested via a cross-validated linear discriminant analysis (CV-LDA) using the package MASS (Ripley et al. 2013). Statistical analyses have been done in R (R Core Team 2022). Conventional LDA and backward stepwise method was used to create an easy-to-use numeric key for separating species *via* character reduction. Data input for this analysis was performed as absolute measurements (raw data).

### ﻿Imaging (BS)

Z.stack images of mounted ants were produced with Keyence a VHX 7000 digital microscope using the multi-lightning mode at magnifications between 80× and 400×.

## ﻿Results

**Figure 3.**
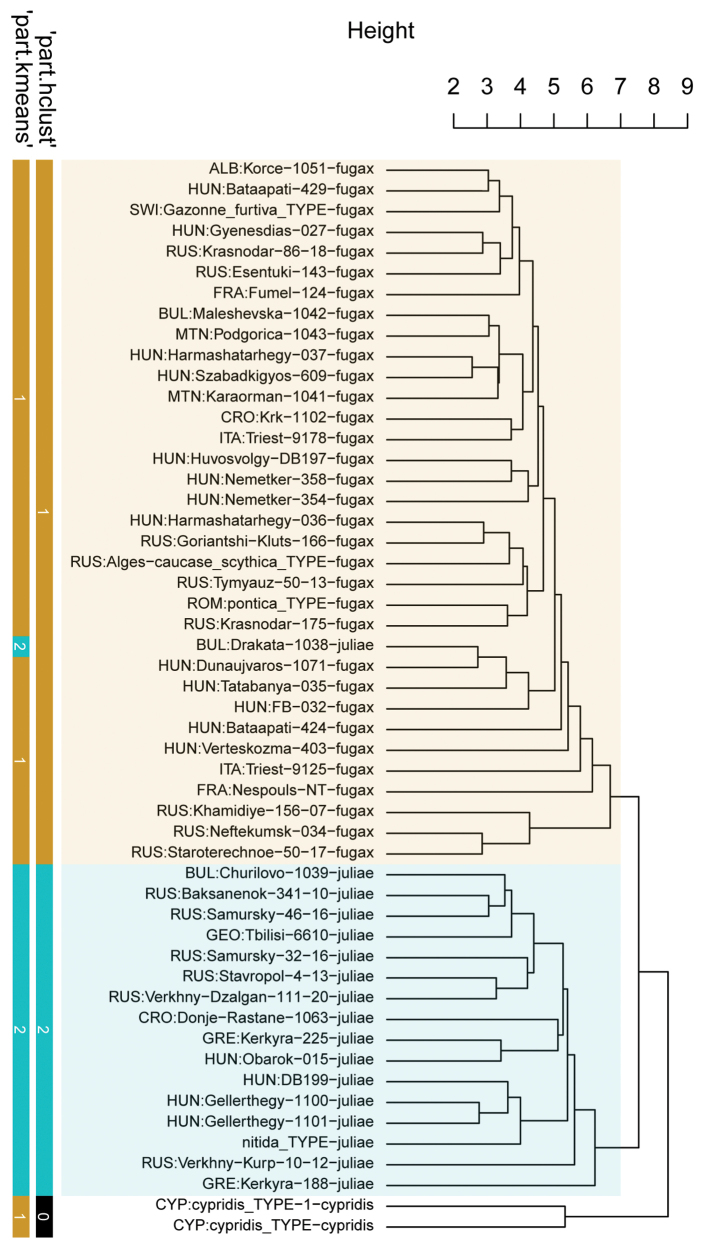
Dendrogram comparing the results of “kmeans”, and “hclust” in NC Clustering using UPGMA distance method of *Solenopsis* workers’ morphometric raw data. Ocher bars: *S.fugax*, blue bars: *S.juliae*. The type material of *S.juliae* was not available for measurements, not shown. Black bar represents the *S.cypridis* samples as outgroup. Data input: raw data within the character system of SC. The *cypridis* cluster (black bars) was assigned as outlier in ‘hclust’ but ‘kmeans’ assigned it in the *fugax* cluster. Note: four workers mounted on different pins (3 and 1 workers respectively) of the *S.cypridis* syntype material appear separately in the tree.

**Figure 4.**
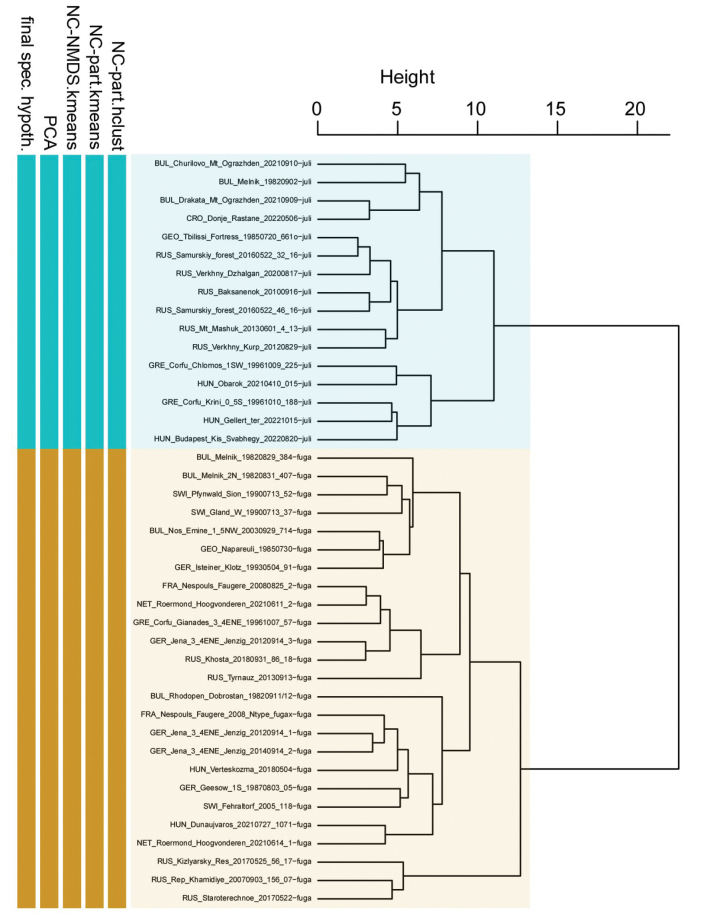
Comparison of five exploratory data analyses with the final species hypothesis. Dendrogram showing NC Clustering using ward distance method. Ocher bars: *Solenopsisfugax*, blue bars: *S.juliae*. Data input: RAV-corrected values of workers in the character system of BS.

**Figure 5.**
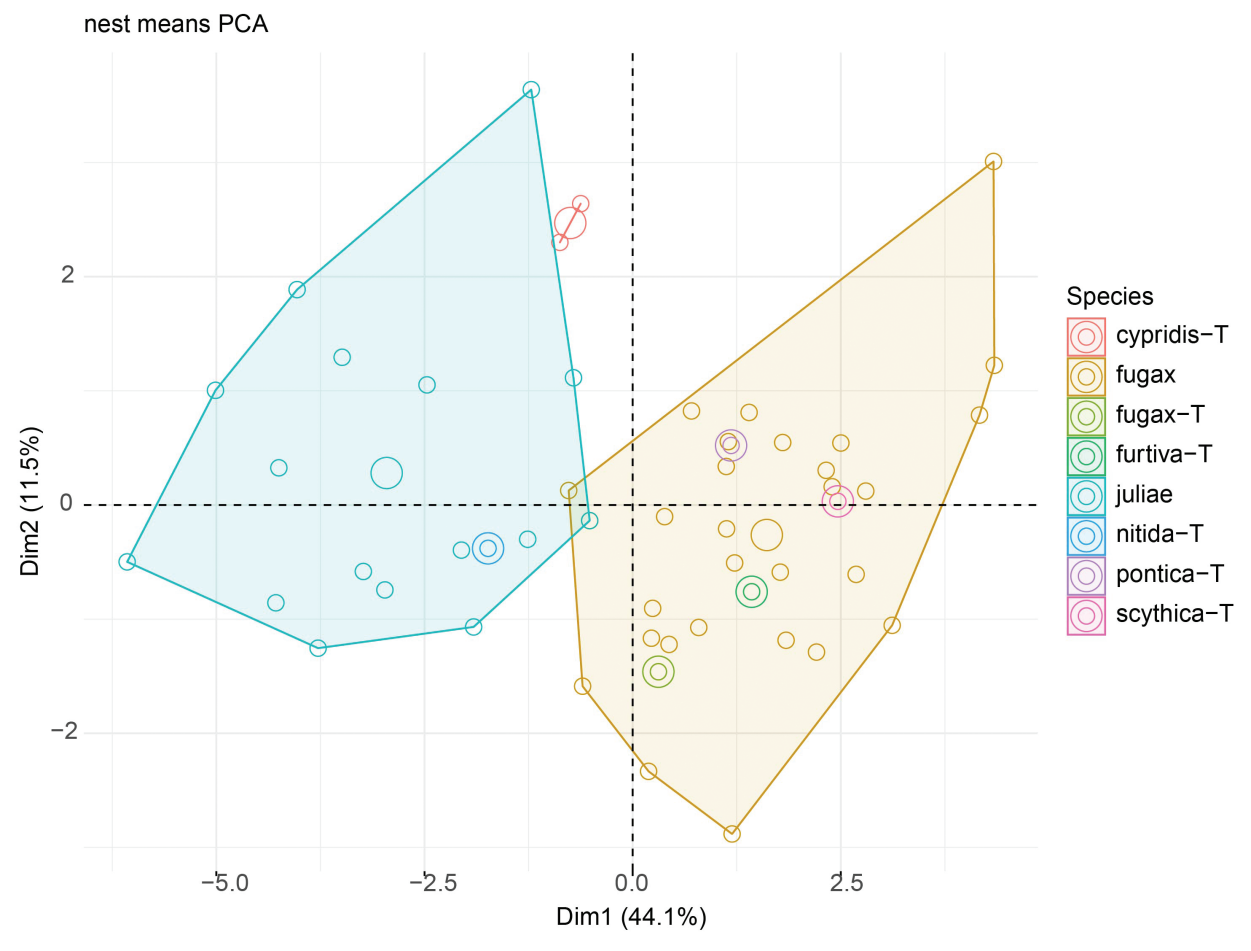
Principal component analyses of residuals of *Solenopsis* worker morphometric data. Each small dot represents a colony sample. Large dots represent centroids. Double rings represent type specimens or type series. Note: four workers mounted on two different pins (3 and 1 workers respectively) of the *S.cypridis* syntype material appear separately in the plot as red circles.

**Figure 6.**
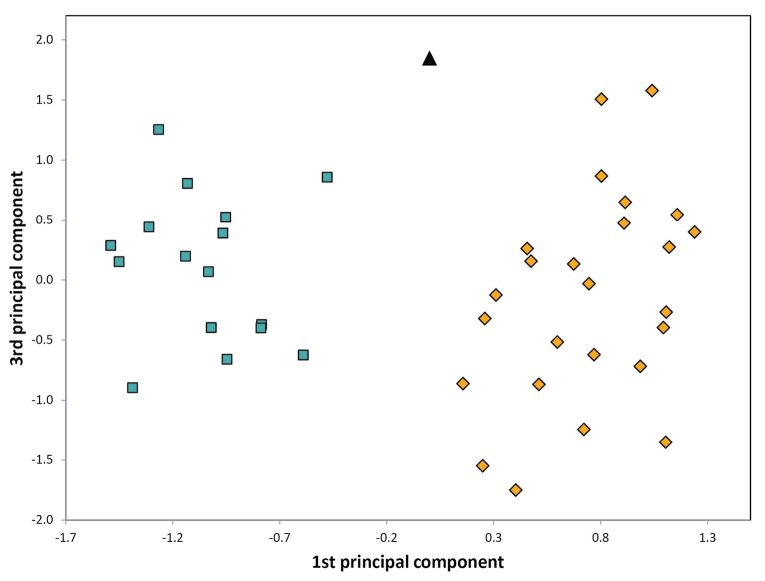
Principal component analyses of RAV data of *Solenopsis* workers. Each dot represents a colony sample. Blue rectangles: *S.juliae*, ocher diamonds: *S.fugax*, black triangles: *S.cypridis*.

**Figure 7.**
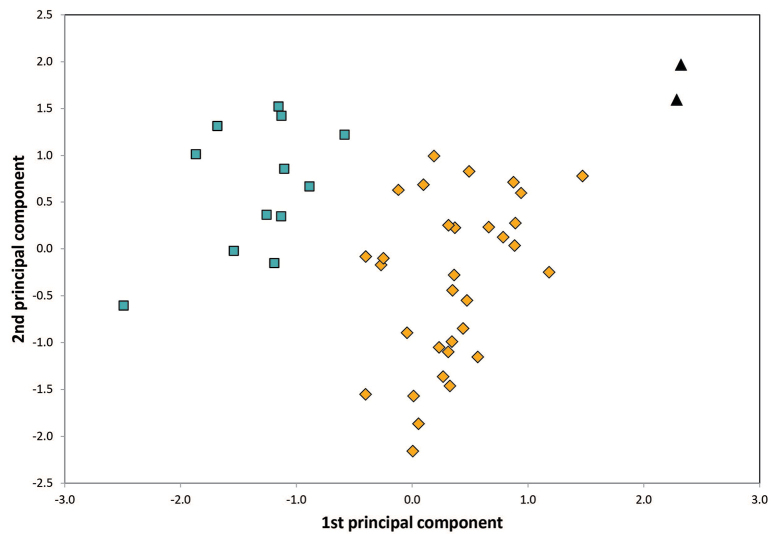
Principal component analyses of RAV data of *Solenopsis* queen individuals. Blue rectangles: *S.juliae*, ocher diamonds: *S.fugax*, black triangles: *S.cypridis*.

The two clustering methods ‘hclust’ and ‘kmeans’ of PART (SC and BS) and NC-NMDS.kmeans (BS only) in combination with NC-clustering resulted in two clusters. The partitioning methods returned a single alternative assignment out of the total 52 (SC, Fig. 3), and no misplacement was returned in BS’ dataset (Fig. 4). The misplaced sample (in SC) was set to wild-card in the confirmatory LDA (Seifert et al. 2014). The syntypes series of Solenopsisfugaxvar.cypridis Santschi, 1934 formed an outlier cluster. The PCA after removal of allometric variance (RAV) according to the approach of SC corroborated the pattern returned by the NC-PART-clustering: two clusters are recognized, and the syntype series of Solenopsisfugaxvar.cypridis Santschi, 1934 is not nested in either cluster (Fig. 5). The outlying placement of the worker and gyne syntype series of *S.cypridis* is confirmed by the PCA using the character set and the RAV correction according to system of BS (Figs 6, 7).

Classifications of the type materials investigated in workers are as follows (Figs 3–7): Cluster A: *Solenopsisnitida* (Dlussky & Radchenko, 1995) and Cluste B: Neotype series of *Solenopsisfugax* (Latreille, 1798), Solenopsis (Diplorhoptrum) fugax var. furtiva Santschi, 1934, Solenopsis (Diplorhoptrum) fugax var. pontica Santschi, 1934, Solenopsis (Diplorhoptrum) fugax var. scytica Santschi, 1934.

The cross-validation LDA confirmed this classification on individual level with 98% probability involving all characters (Table 3), and the high divergence of morphological clusters allows for significant character reduction in creating an easy-to-use numeric key using backward stepwise method in LDA. The three best selected characters returned 96.5% separation between the individuals of the two species, *S.fugax* and *S.juliae*: D3 = 0.060*ML -0.047*CWb -0.125*CLYW-4.582, D3fugax (*n* = 137) = +1.713 [-1.722, +4.954], and D3juliae (*n* = 62) = -1.713 [-4.698, +0.454]

**Table 3.** Cross-validation table for *S.fugax* and *S.juliae* individuals.

	*S.fugax*	*S.juliae*	Percentage correct
*S.fugax*	134	3	97.8
*S.juliae*	1	61	98.4

The same function yields non-overlapping range of scores if nest samples are considered:

D3fugax (*n* = 33) = +1.776 [+0.931, +3.374] and D3juliae (*n* = 17) = -1.678 [-3.477, +0.010].

Investigation of gynes according to the character system of BS confirmed the results obtained in workers. There is a placement of the two syntype gynes of *S.cypridis* clearly separate from the well-separated clusters of *S.fugax* and *S.juliae* in a PCA (Fig. 7).

### ﻿Taxonomic treatment by species

The main sources for identification of a taxon are given in square brackets after taxonomic name, author and year.

#### ﻿ Solenopsis fugax

Taxon classificationAnimaliaAsteralesCampanulaceae

(Latreille, 1798)

8DA6ECB4-B1C6-5D90-BFB8-6C8756E2E7BB

Formica fugax Latreille, 1798. [type investigation]

##### Notes.

The species was described from the environs of Brive, France. Five workers and two gynes were investigated from the neotype nest sample, labelled “FRA: 45.0517°N, 1.5372°E, Nespouls-Faugère. 330 m, sous une pierre, leg. Galkowski 25. VIII. 2008 -1”, “Neoparatypes of *Solenopsisfugax* (Latreille, 1798) des. Galkowski, Casewitz-Weulersse et Cagniant 2010”, depository Senckenberg Museum of Natural History Görlitz. The clear placement of the worker and gyne type specimens within the *S.fugax* cluster has been shown in the analyses above.

#### ﻿ Solenopsis flavidula

Taxon classificationAnimaliaAsteralesCampanulaceae

(Nylander, 1849)

0F9F5BBA-3654-539C-97D4-DE1759A4D420

Myrmica flavidula Nylander, 1849. [type investigation]

##### Notes.

The species was described from the region of the Don river, southern Russia. Seven syntypes from FMNH Helsinki were investigated: two workers on one pin, “Ross. merid.”, “Motchoulsky”, “Coll. Nylandr.”, “Mus. Zool. H: fors Spec. typ. No. 5107 *Myrmicaflavidula* Nyl.”; five workers on three pins with the same labels as the previous, but type Nos. 5107, 5108, and 5109. Two type specimens allowed the recording of the full set of 18 characters in the measuring system of BS. The synonymy was confirmed by a wild-card run in a LDA which allocated the two type specimens with p = 0.9996 to the *S.fugax* cluster.

#### ﻿ Solenopsis (Diplorhoptrum) fugax var. pontica

Taxon classificationAnimaliaAsteralesCampanulaceae

Santschi, 1934 [type investigation]

7492CD67-4378-5EC4-83C8-14A9BAE46DC4

##### Notes.

This taxon was described from Romania. Two syntype workers were investigated labelled “*Solenopsisfugax*. Latr. v. *pontica* Sants” “MOLDAVIE, VALL. DU BERLAD, A. L. Montandon”, “ANTWEB CASENT 0913885”; depository NHM Basel. The placement of the worker type specimens within the *S.fugax* cluster has been shown in the analyses above.

#### ﻿ Solenopsis (Diplorhoptrum) fugax var. scytica

Taxon classificationAnimaliaAsteralesCampanulaceae

Santschi, 1934 [type investigation]

C2C9DF8C-CF1D-57AC-9B6B-5005B7E29CC5

##### Notes.

This taxon was described from the Great Caucasus. Two syntype workers were investigated labelled “*Solenopsisfugax*. Latr. v. *scythica* Sant”, “Alages Caucase, Mejunoff.”, “ANTWEB CASENT 0913886”; depository NHM Basel. The placement of the worker type specimens within the *S.fugax* cluster has been shown in the analyses above.

#### ﻿ Solenopsis (Diplorhoptrum) fugax var. furtiva

Taxon classificationAnimaliaAsteralesCampanulaceae

Santschi, 1934 [type investigation]

42709A0A-A42F-5E44-B151-49A04DC06E2C

##### Notes.

This taxon was described from France. Five syntype workers were examined labelled “Hte Garonne, Mt d’Espinasse, Val.Larboust, 1250 m. 03.X.1929”; depository NHM Basel. The placement of the worker type specimens within the *S.fugax* cluster has been shown in the analyses above.

##### Material examined.

Numeric phenotypical data were taken by SC in 35 samples with 137 workers. BS investigated 25 nest samples with 92 workers and 32 gynes, the latter collected either from nests or caught during nuptial flight or in traps. For details see Suppl. materials 2, 3.

##### Geographic range.

**Figure 8.**
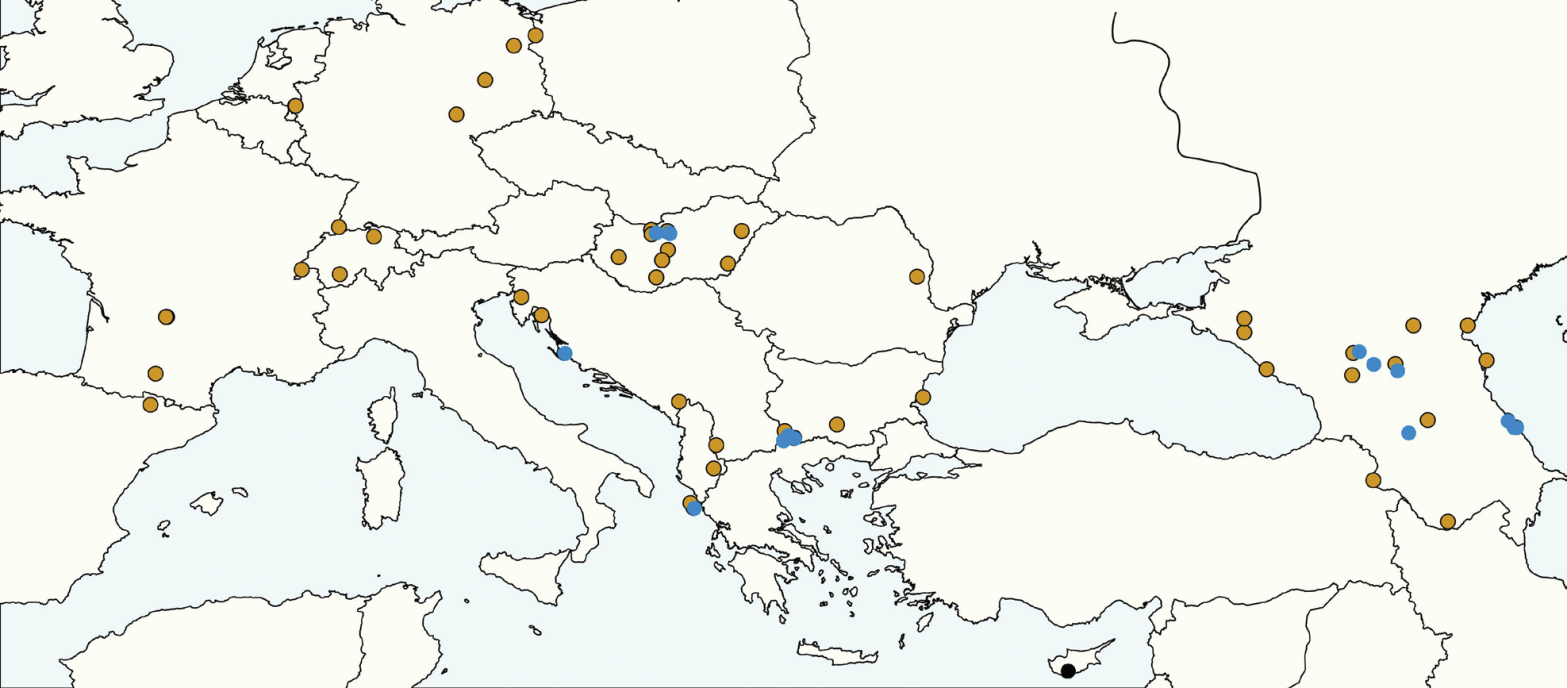
Geographic map shows the distribution of the samples analyzed in this study. Blue dots: *S.juliae*, ocher dots: *S.fugax*, black dots: *S.cypridis*.

According to our data ranging from France over Central Europe, the Balkans, Asia Minor, the Caucasian region, the south Ukrainian and Russian steppes east to the Caspian Sea (Fig. 8). A wider range including Spain and stretching east to east Kazakhstan appears very likely but is not documented so far by examined vouchers.

##### Diagnoses.

**Figure 9.**
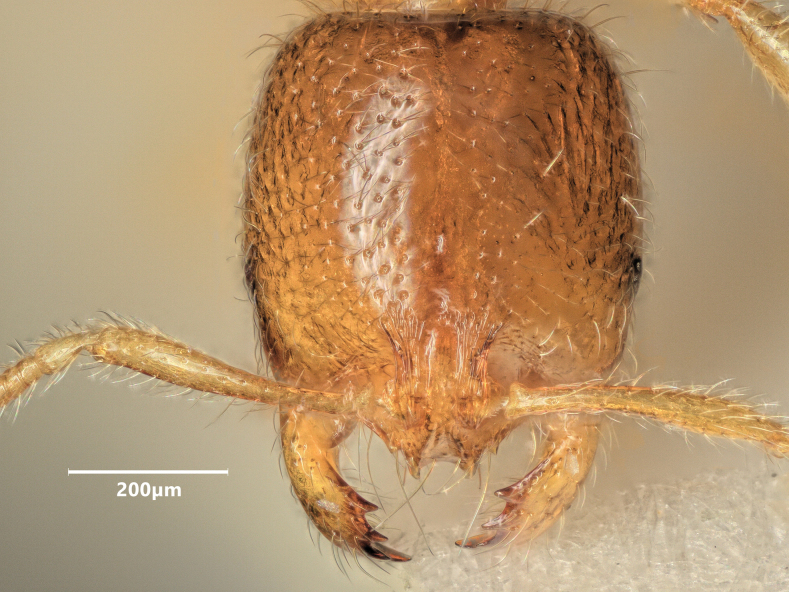
Head of major worker of *Solenopsisfugax* in full-face view.

**Figure 10.**
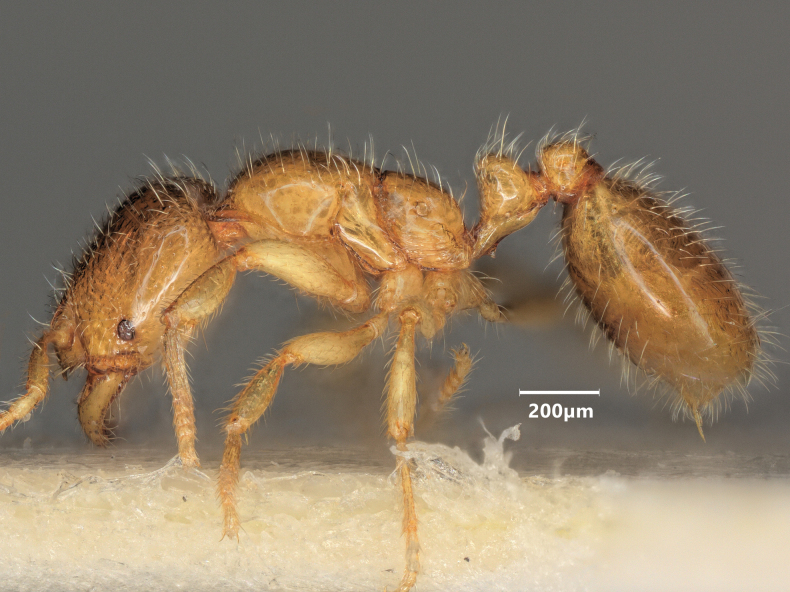
Lateral view of *Solenopsisfugax* major worker.

**Figure 11.**
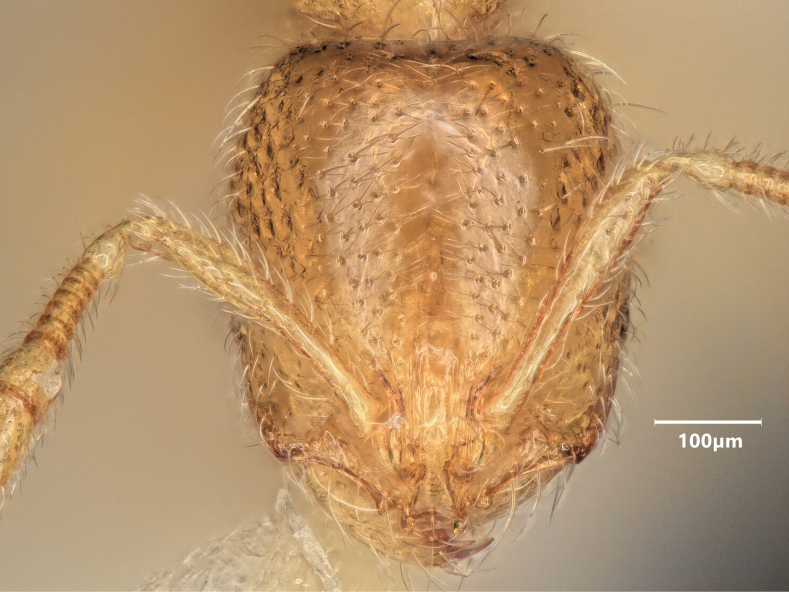
Head of minor worker of *Solenopsisfugax* in full-face view.

**Figure 12.**
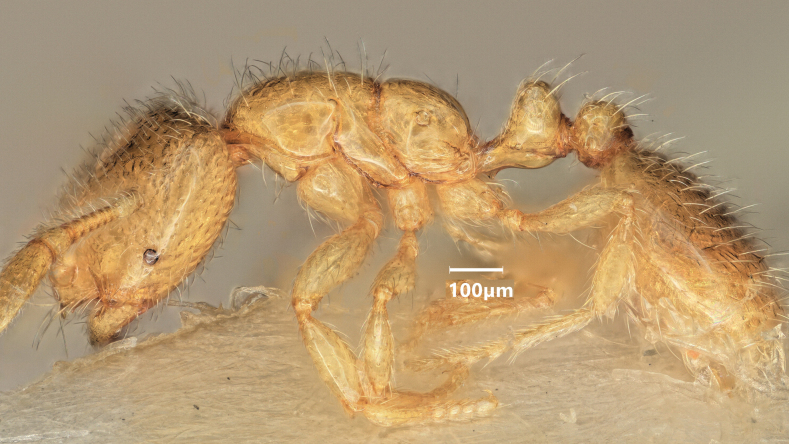
Lateral view of *Solenopsisfugax* minor worker.

***Worker*** (Table 4, Figs 9–12): Small, mean CS 491 µm. All shape ratios given below are mean values allometrically corrected for CS = 480 µm. Head elongated, CL/CW_480_ 1.184. Hind margin of vertex in full face view both in minors and majors straight or very feebly concave. Scape short, SL/CS_480_ 0.705. Frontal carinae short, often slightly diverging frontad, FL/CS_480_ 0.231, FR/CS_480_ 0.221. Preocular distance rather large, PrOc/CS_480_ 0.191. Eye small, EL/CS_480_ 0.091. Inner clypeal dents spiny and moderately long (CLSPLM/CS_480_ 0.058), their tips often slightly incurving and as result more approached (CLSPD/CS_480_ 0.123). Lateral clypeal dents much less developed (CLSPLL/CS_480_ 0.021). Frontal lobes carinulate, whole surface vertex except for numerous foveolae of the seta bases and occasional vestigial microrugulae completely smooth and shiny. Mesosoma long (ML/CS_480_ 1.214), moderately wide (MW/CS_480_ 0.601) and always with a moderately deep metanotal groove (MpGr/CS_480_ 0.028). Whole mesosoma smooth and shiny except for 3–6 longitudinal carinulae on lateral metapleuron. Petiole in lateral view with a relatively short peduncle and a high node with a semicircular dorsum, the whole node slightly inclined caudad. Petiole much higher and only slightly narrower than postpetiole (PEH/CS_480_ 0.388, PPH/CS_480_ 0.303, PEW/CS_480_ 0.320, PPW/CS_480_ 0.333). Both waist segments completely smooth and shiny. Head, mesosoma, waist, gaster, femora, tibiae, and scape with very abundant, fine and long setae. Pubescence absent. Whole body and appendages light yellowish, larger major workers often with a brownish color component.

**Table 4.** Morphometric data of *Solenopsis* workers in the arrangement of arithmetic mean ± standard deviation [lower extreme, upper extreme].

Primary indices				Indices after removal of allometric variance, adjusted for CS = 480 µm			
	*S.cypridis* (types, *n* = 4)	*S.fugax* (*n* = 92)	*S.juliae* (*n* = 66)		*S.cypridis* (types, *n* = 4)	*S.fugax* (*n* = 92)	*S.juliae* (*n* = 66)
CS [µm]	515 ± 38 [462, 552]	491 ± 67 [377, 660]	460 ± 48 [385, 560]	CS [µm]	515 ± 38 [462, 552]	491 ± 67 [377, 660]	460 ± 48 [385, 560]
CL/CW	1.143 ± 0.036 [1.110, 1.182]	1.177 ± 0.044 [1.068, 1.261]	1.163 ± 0.034 [1.084, 1.242]	CL/CW_480_	1.159 ± 0.028 [1.137, 1.200]	1.184 ± 0.022 [1.136, 1.238]	1.152 ± 0.020 [1.102, 1.200]
SL/CS	0.691 ± 0.010 [0.679, 0.700]	0.703 ± 0.016 [0.659, 0.738]	0.695 ± 0.015 [0.652, 0.731]	SL/CS_480_	0.696 ± 0.007 [0.688, 0.705]	0.705 ± 0.014 [0.666, 0.739]	0.692 ± 0.013 [0.659, 0.717]
FL/CS	0.230 ± 0.010 [0.219, 0.241]	0.231 ± 0.010 [0.203, 0.257]	0.218 ± 0.007 [0.204, 0.237]	FL/CS_480_	0.230 ± 0.009 [0.219, 0.241]	0.231 ± 0.010 [0.203, 0.258]	0.218 ± 0.007 [0.205, 0.237]
FR/CS	0.230 ± 0.010 [0.219, 0.241]	0.221 ± 0.009 [0.198, 0.244]	0.209 ± 0.008 [0.190, 0.237]	FR/CS_480_	0.230 ± 0.009 [0.219, 0.241]	0.221 ± 0.009 [0.199, 0.242]	0.210 ± 0.008 [0.189, 0.237]
EL/CS	0.098 ± 0.014 [0.085, 0.113]	0.092 ± 0.011 [0.064, 0.124]	0.095 ± 0.007 [0.082, 0.116]	EL/CS_480_	0.095 ± 0.011 [0.082, 0.106]	0.091 ± 0.010 [0.060, 0.125]	0.096 ± 0.005 [0.081, 0.109]
PrOc/CS	0.199 ± 0.013 [0.187, 0.217]	0.191 ± 0.010 [0.169, 0.214]	0.185 ± 0.008 [0.168, 0.202]	PrOc/CS_480_	0.200 ± 0.012 [0.188, 0.216]	0.191 ± 0.010 [0.170, 0.214]	0.184 ± 0.008 [0.168, 0.201]
CLSPLM /CS	0.066 ± 0.004 [0.059, 0.069]	0.058 ± 0.006 [0.046, 0.072]	0.055 ± 0.006 [0.040, 0.070]	CLSPLM /CS_480_	0.067 ± 0.005 [0.060, 0.071]	0.058 ± 0.005 [0.043, 0.073]	0.055 ± 0.006 [0.040, 0.069]
CLSPLL /CS	0.021 ± 0.005 [0.016, 0.027]	0.021 ± 0.006 [0.007, 0.036]	0.019 ± 0.004 [0.011, 0.028]	CLSPLL /CS_480_	0.021 ± 0.005 [0.017, 0.027]	0.021 ± 0.006 [0.007, 0.041]	0.018 ± 0.004 [0.011, 0.030]
CLSPD /CS	0.157 ± 0.015 [0.142, 0.174]	0.124 ± 0.014 [0.091, 0.154]	0.146 ± 0.010 [0.120, 0.168]	CLSPD /CS_480_	0.154 ± 0.014 [0.137, 0.170]	0.123 ± 0.012 [0.093, 0.149]	0.148 ± 0.009 [0.128, 0.164]
ML/CS	1.186 ± 0.024 [1.164, 1.216]	1.214 ± 0.029 [1.133, 1.303]	1.155 ± 0.019 [1.104, 1.186]	ML/CS_480_	1.186 ± 0.024 [1.164, 1.216]	1.214 ± 0.029 [1.133, 1.303]	1.155 ± 0.019 [1.104, 1.185]
MW/CS	0.596 ± 0.004 [0.591, 0.600]	0.600 ± 0.012 [0.577, 0.631]	0.587 ± 0.012 [0.565, 0.617]	MW/CS_480_	0.600 ± 0.005 [0.595, 0.606]	0.601 ± 0.013 [0.573, 0.637]	0.585 ± 0.010 [0.565, 0.612]
MpGr /CS	0.034 ± 0.005 [0.027, 0.038]	0.028 ± 0.009 [0.009, 0.058]	0.027 ± 0.005 [0.014, 0.039]	MpGr /CS_480_	0.035 ± 0.005 [0.028, 0.040]	0.028 ± 0.009 [0.009, 0.058]	0.027 ± 0.005 [0.014, 0.039]
PEW/CS	0.313 ± 0.009 [0.307, 0.326]	0.321 ± 0.014 [0.279, 0.356]	0.295 ± 0.014 [0.269, 0.334]	PEW/CS_480_	0.311 ± 0.009 [0.304, 0.324]	0.320 ± 0.014 [0.281, 0.351]	0.296 ± 0.014 [0.268, 0.336]
PPW/CS	0.320 ± 0.009 [0.313, 0.332]	0.333 ± 0.012 [0.301, 0.373]	0.326 ± 0.013 [0.289, 0.366]	PPW/CS_480_	0.322 ± 0.010 [0.313, 0.335]	0.333 ± 0.013 [0.306, 0.371]	0.324 ± 0.012 [0.290, 0.362]
PEH/CS	0.369 ± 0.016 [0.353, 0.390]	0.386 ± 0.013 [0.354, 0.418]	0.371 ± 0.015 [0.343, 0.402]	PEH/CS_480_	0.375 ± 0.011 [0.364, 0.387]	0.388 ± 0.011 [0.361, 0.411]	0.368 ± 0.012 [0.341, 0.394]
PPH/CS	0.307 ± 0.015 [0.295, 0.328]	0.307 ± 0.013 [0.272, 0.369]	0.297 ± 0.010 [0.270, 0.319]	PPH/CS_480_	0.307 ± 0.015 [0.296, 0.328]	0.303 ± 0.013 [0.268, 0.364]	0.292 ± 0.010 [0.266, 0.314]

**Figure 13.**
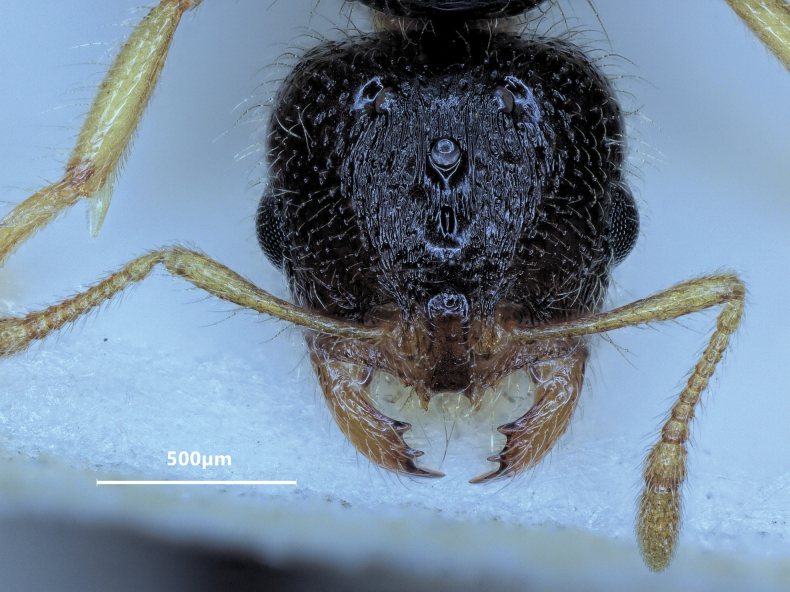
Head of a macrogyne of *Solenopsisfugax* in full-face view.

**Figure 14.**
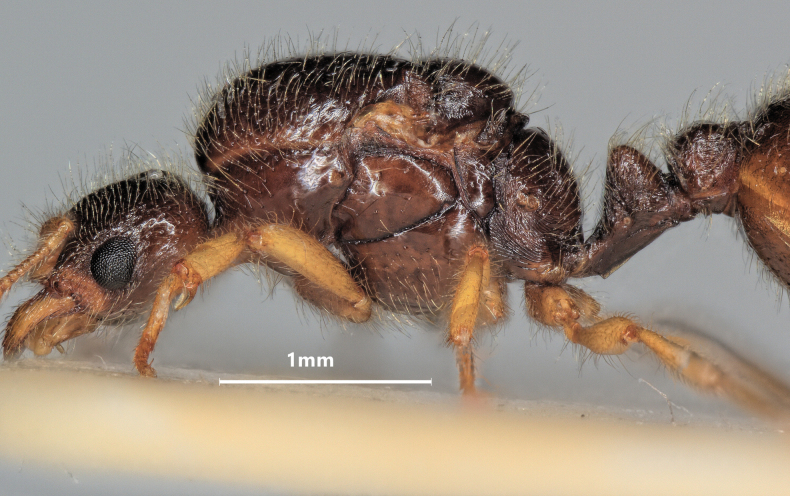
Lateral view of a *Solenopsisfugax* macrogyne.

**Figure 15.**
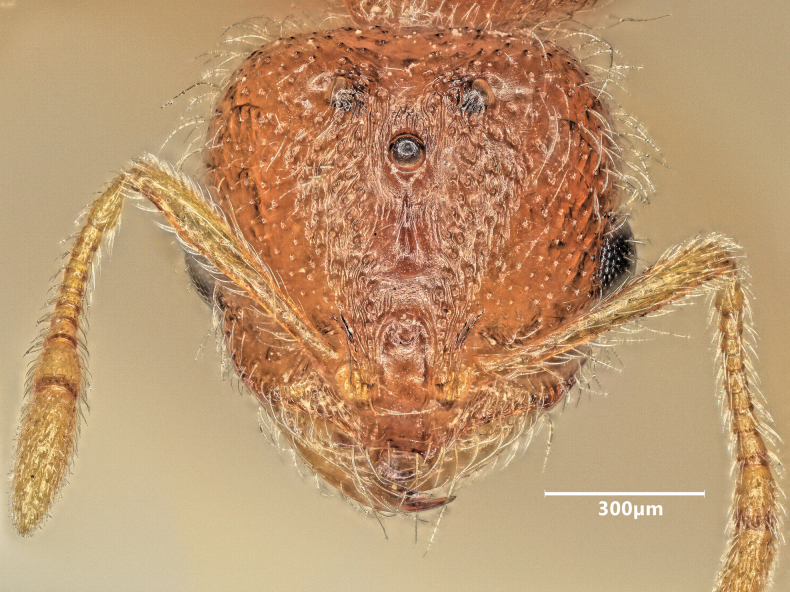
Head of a microgyne of *Solenopsisfugax* in full-face view.

***Gyne*** (Table 5, Figs 13–15): Medium-sized, mean CS 897 µm. Head clearly shorter than wide, CL/CW 0.939. Inner third of hind margin of vertex in full face view weakly convex or straight. Scape short, SL/CS 0.637. Frontal carinae short, subparallel and moderately distant, FR/CS 0.252. Preocular distance small, PrOc/CS 0.119. Eye moderately large, EL/CS 0.287. Inner clypeal dents spiny and rather long (CLSPLM/CS 0.050), rather approached (CLSPD/CS 0.141). Lateral clypeal dents much less developed (CLSPLL/CS 0.015). Genae and frontal lobes carinulate, central vertex strongly carinulate-rugulose; these characters are also found in the two microgynes corresponding in size to the *S.juliae* macrogynes. Mesosoma long (ML/CS 2.072) and much higher than wide (MH/CS 1.315, MW/CS 1.117). Dorsal mesonotum and scutum moderately carinulate. Lateral scutum, metapleuron and lower propodeum more strongly carinulate. Petiole in lateral view with a short peduncle, a high node with triangular profile and slightly higher than postpetiole (PEH/CS 0.493, PPH/CS 0.479); dorsal crest of petiole node in dorsal view much wider than long, the petiole slightly narrower than postpetiole (PEW/CS 0.463, PPW/CS 0.513). Postpetiole densely but weakly carinulate. Head, mesosoma, waist, gaster, femora, tibiae, and scape with very abundant, fine and long setae. Pubescence absent. Usually dark to medium brown; legs, antennae and mandibles yellowish.

**Table 5.** Morphometric data of *Solenopsis* gynes in the arrangement of arithmetic mean ± standard deviation [lower extreme, upper extreme].

Primary indices				Indices after removal of allometric variance, adjusted for CS = 850 µm			
	*S.cypridis* types, *n* = 2	*S.fugax* (*n* = 32)	*S.juliae* (*n* = 12)		*S.cypridis* types, *n* = 2	*S.fugax* (*n* = 32)	*S.juliae* (*n* = 12)
CS [µm]	972 ± 4 [969, 975]	897 ± 29 [818, 941]	798 ± 11 [777, 814]	CS [µm]	972 ± 4 [969, 975]	897 ± 29 [818, 941]	798 ± 11 [777, 814]
CL/CW	0.890 ± 0.008 [0.884, 0.895]	0.939 ± 0.026 [0.895, 0.988]	0.969 ± 0.011 [0.953, 0.988]	CL/CW_850_	0.924 ± 0.007 [0.919, 0.928]	0.952 ± 0.024 [0.912, 1.004]	0.953 ± 0.012 [0.937, 0.975]
SL/CS	0.630 ± 0.012 [0.622, 0.639]	0.637 ± 0.015 [0.602, 0.667]	0.657 ± 0.013 [0.630, 0.678]	SL/CS_850_	0.664 ± 0.011 [0.656, 0.672]	0.650 ± 0.014 [0.620, 0.676]	0.643 ± 0.013 [0.618, 0.666]
FL/CS	0.281 ± 0.003 [0.279, 0.283]	0.253 ± 0.011 [0.233, 0.282]	0.245 ± 0.010 [0.224, 0.267]	FL/CS_850_	0.302 ± 0.002 [0.301, 0.304]	0.260 ± 0.010 [0.244, 0.291]	0.237 ± 0.009 [0.218, 0.258]
FR/CS	0.281 ± 0.003 [0.279, 0.283]	0.252 ± 0.009 [0.233, 0.264]	0.240 ± 0.008 [0.221, 0.250]	FR/CS_850_	0.302 ± 0.002 [0.300, 0.303]	0.259 ± 0.008 [0.244, 0.273]	0.233 ± 0.008 [0.214, 0.243]
EL/CS	0.304 ± 0.004 [0.301, 0.307]	0.287 ± 0.010 [0.271, 0.312]	0.295 ± 0.011 [0.278, 0.312]	EL/CS_850_	0.324 ± 0.004 [0.321, 0.326]	0.294 ± 0.009 [0.276, 0.315]	0.287 ± 0.010 [0.273, 0.301]
PrOc/CS	0.126 ± 0.000 [0.126, 0.126]	0.119 ± 0.008 [0.102, 0.134]	0.125 ± 0.007 [0.115, 0.138]	PrOc/CS_850_	0.115 ± 0.000 [0.115, 0.115]	0.115 ± 0.008 [0.101, 0.129]	0.131 ± 0.007 [0.119, 0.144]
CLSPLM /CS	0.055 ± 0.004 [0.052, 0.058]	0.050 ± 0.006 [0.037, 0.063]	0.050 ± 0.006 [0.037, 0.060]	CLSPLM /CS_850_	0.053 ± 0.004 [0.050, 0.056]	0.049 ± 0.006 [0.036, 0.061]	0.050 ± 0.006 [0.037, 0.061]
CLSPLL /CS	0.014 ± 0.001 [0.013, 0.014]	0.015 ± 0.004 [0.007, 0.024]	0.012 ± 0.003 [0.005, 0.017]	CLSPLL /CS_850_	0.009 ± 0.001 [0.009, 0.009]	0.013 ± 0.003 [0.006, 0.019]	0.016 ± 0.005 [0.006, 0.023]
CLSPD /CS	0.174 ± 0.008 [0.168, 0.180]	0.141 ± 0.008 [0.126, 0.157]	0.162 ± 0.011 [0.145, 0.185]	CLSPD /CS_850_	0.174 ± 0.008 [0.168, 0.179]	0.141 ± 0.008 [0.126, 0.157]	0.162 ± 0.011 [0.145, 0.185]
MW/CS	1.154 ± 0.018 [1.142, 1.167]	1.117 ± 0.048 [1.043, 1.257]	1.016 ± 0.030 [0.962, 1.080]	MW/CS_850_	1.069 ± 0.013 [1.059, 1.078]	1.083 ± 0.042 [1.004, 1.189]	1.052 ± 0.034 [0.994, 1.134]
MH/CS	1.334 ± 0.055 [1.295, 1.373]	1.315 ± 0.043 [1.240, 1.397]	1.248 ± 0.048 [1.175, 1.330]	MH/CS_850_	1.250 ± 0.054 [1.212, 1.289]	1.276 ± 0.038 [1.214, 1.358]	1.270 ± 0.054 [1.191, 1.357]
ML/CS	2.093 ± 0.042 [2.064, 2.123]	2.072 ± 0.050 [1.958, 2.163]	1.991 ± 0.048 [1.912, 2.085]	ML/CS_850_	2.026 ± 0.038 [1.999, 2.053]	2.046 ± 0.046 [1.929, 2.124]	2.020 ± 0.053 [1.939, 2.127]
PEW/CS	0.490	0.463 ± 0.026 [0.401, 0.527]	0.421 ± 0.017 [0.384, 0.452]	PEW/CS_850_	0.510	0.470 ± 0.026 [0.403, 0.534]	0.414 ± 0.016 [0.378, 0.442]
PPW/CS	0.565	0.513 ± 0.029 [0.449, 0.565]	0.496 ± 0.025 [0.440, 0.526]	PPW/CS_850_	0.604	0.526 ± 0.029 [0.465, 0.579]	0.482 ± 0.024 [0.428, 0.510]
PEH/CS	0.494 ± 0.014 [0.484, 0.503]	0.493 ± 0.020 [0.451, 0.529]	0.476 ± 0.018 [0.450, 0.505]	PEH/CS_850_	0.481 ± 0.013 [0.472, 0.490]	0.488 ± 0.019 [0.449, 0.526]	0.482 ± 0.018 [0.455, 0.512]
PPH/CS	0.515 ± 0.010 [0.508, 0.522]	0.479 ± 0.023 [0.417, 0.522]	0.470 ± 0.020 [0.429, 0.492]	PPH/CS_850_	0.528 ± 0.017 [0.516, 0.540]	0.483 ± 0.023 [0.422, 0.527]	0.465 ± 0.019 [0.424, 0.484]

#### ﻿ Solenopsis juliae

Taxon classificationAnimaliaAsteralesCampanulaceae

(Arakelian, 1991)

822E588D-3BA4-5A49-B1BB-549DCA1823C1

Diplorhoptrum juliae Arakelian, 1991. [description]

##### Notes.

The species was described from Armenia. The type series was collected by G. Arakelian in a clearing of an oak forest at 1750 m near to the village Arzakan [40.450°N, 44.608°E], 30 August 1988 (Arakelian 1991). The ongoing Russian-Ukrainian war prevents a direct examination of type specimens deposited in the museums of Erevan, Kiev, and Moscow but the original description allows a fairly good conclusion that *S.juliae* is the oldest available name for our eastern species. This is based on three arguments: (1) using a conversion factor of 1.148 for Arakelian’s “head width before eyes” given by him as 0.72 mm, the holotype gyne has a CW of 0.827 mm; (2) the ratio CLSPD/CW in the holotype gyne taken from the drawing is 0.169. This results in a CLSPD of 0.140 mm; (3) Arakelian reported a reduced sculpture: “Body largely smooth and shiny. A weak superficial sculpture is notable on head sides, frontal lobes, around the antennal scrobes, on propodeum and waist”. This means the absence of a notable sculpture on central vertex. This is precisely what we found as a character separating it from *S.fugax*. A discriminant 35.24*CW-58.91*CLSPD-22.59 provides a full separation of the 12 measured *S.juliae* and 32 measured *S.fugax* gynes with the former species ranging between -2.73 and -0.47 and the latter between +0.47 and +4.32. Run as wild-card, the holotype gyne of *S.juliae* scores -1.69 meaning a posterior probability of 0.9999. A synonymy of *S.juliae* with *S.ilinei* Santschi, 1936 and *S.deserticola* Ruzsky, 1905 is excluded by the much smaller ratio CL/CW.

#### ﻿ Solenopsis nitida

Taxon classificationAnimaliaAsteralesCampanulaceae

(Dlussky & Radchenko, 1994)

ED51D288-4E08-505C-9447-DD3D31E77D61

Diplorhoptrum nitidum Dlussky & Radchenko, 1994. [images of types, description]

##### Notes.

This taxon was described also from Armenia, some 200 km SE of the type locality of *S.juliae*. The type series consists of workers and gynes collected by A. Radchenko from a single nest near to the village Legvaz [38.938°N, 46.216°E], 25 June 1986. Direct examination of type specimens deposited in the museums of Kiev and Moscow was also prevented by the ongoing Russian-Ukrainian war. Instead, we evaluated the images of the holotype gyne and a paratype worker provided by www.antweb.org under the specimen identifiers CASENT 0917366 and CASENT 0917367. The evaluation suggests a synonymy of *Solenopsisnitida* with *S.juliae* based upon the following arguments. The measurements of the paratype worker derivable with an acceptable error from the images were in µm CL 557, CW 493, FL 99, FR 99, CLSPD 89, PEW 163, PPW 156, MW 293 and ML 609. Running the absolute data as wild-card in a LDA against the data pool of BS, the paratype is allocated with p = 0.9994 to the *S.juliae* cluster and it is allocated also to this cluster by the PCA using the data set of SC (Fig. 5). Furthermore, the holotype gyne shows the sculpture reduction characteristic for *S.juliae* but there is one problem: the gyne is, as it is the case in all the paratype gynes, much smaller than average *S.juliae* gynes. As there is no indication for miscalibration of the scale in the images by comparing with measurements given by Dlussky and Radchenko (1994), these small absolute body size values must be considered as real. We derived the following measurements in µm from the images of the holotype gyne: CL 659, CW 668, FL 134, FR 134, CLSPD 100, MW 528, PEH 291, PPH 280, MH 705, and ML 1301. Proposing herewith a synonymy of *S.nitida* with *S.juliae*, we consider the holotype gyne as a microgyne with allometric changes of body shape, but we also encourage a more profound investigation of the problem after extensive collections have been done in the Caucasian region.

##### Material examined.

Numeric phenotypical data were taken by SC in 17 samples (largely nest samples) with 62 workers. BS investigated 16 nest samples with 66 workers and 12 gynes, the latter collected either from nests or caught during nuptial flight. For details see Suppl. materials 1, 2.

##### Geographic range.

According to our data including the Pannonian Basin and the complete Balkans and stretching east over Asia Minor (Fig. 8), the whole Caucasian region to the western shores of the Caspian Sea (Yusupov 2014).

##### Diagnosis.

**Figure 16.**
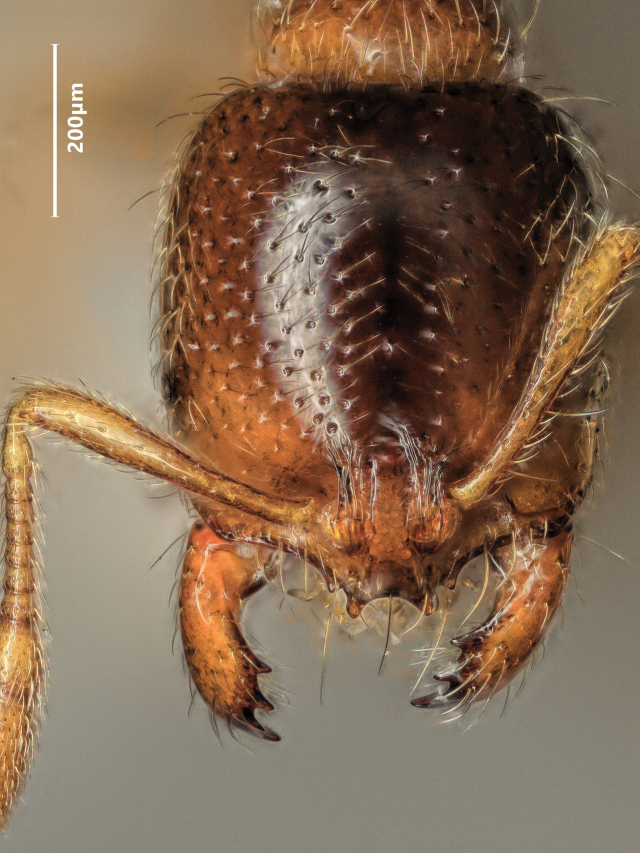
Head of major worker of *Solenopsisjuliae* in full-face view.

**Figure 17.**
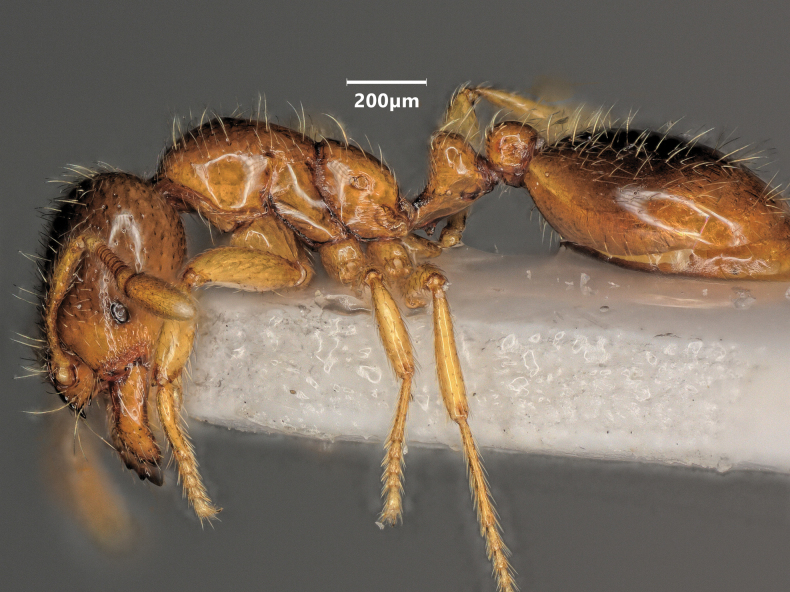
Lateral view of *Solenopsisjuliae* major worker.

**Figure 18.**
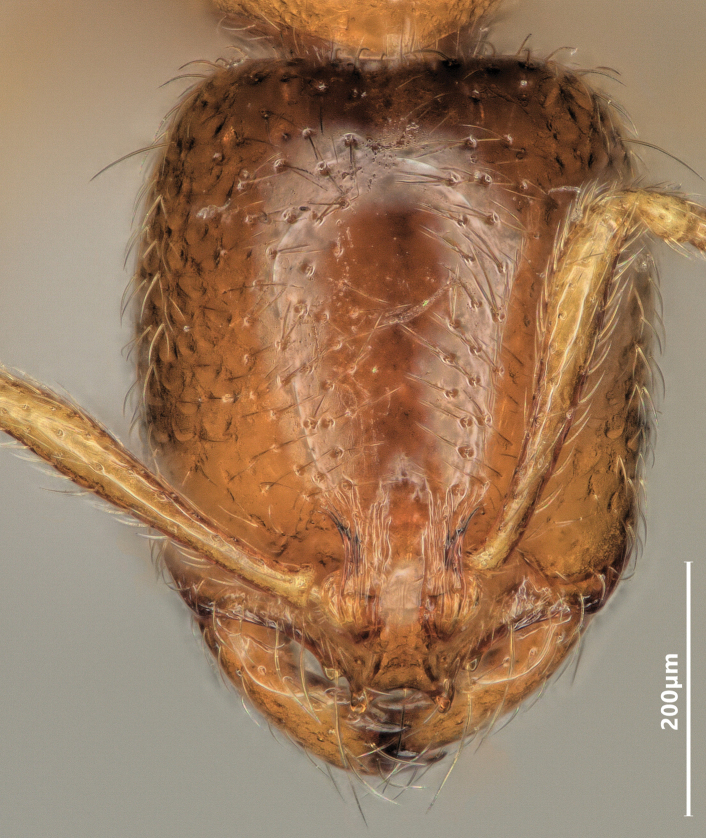
Head of minor worker of *Solenopsisjuliae* in full-face view.

**Figure 19.**
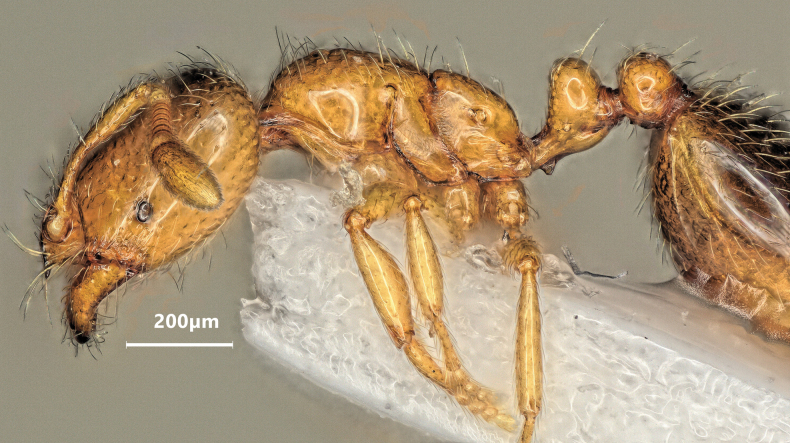
Lateral view of *Solenopsisjuliae* minor worker.

***Worker*** (Table 4, Figs 16–19): Smaller than *fugax*, mean CS 460 µm. All shape ratios given below are mean values allometrically corrected for CS = 480 µm. Head less elongated than in *fugax*, CL/CW_480_ 1.152. Hind margin of vertex in full face view both in minors and majors straight or very feebly concave. Scape slightly shorter than in *fugax*, SL/CS_480_ 0.692. Frontal carinae short, often slightly diverging frontad, FL/CS_480_ 0.218, FR/CS_480_ 0.210. Preocular distance slightly smaller than in *fugax*, PrOc/CS_480_ 0.184. Eye small, EL/CS_480_ 0.096. Inner clypeal dents spiny and moderately long (CLSPLM/CS_480_ 0.055), their tips often diverging and as result more distant than in *fugax* (CLSPD/CS_480_ 0.148). Lateral clypeal dents small (CLSPLL/CS_480_ 0.018). Frontal lobes carinulate, whole surface of vertex except for the numerous foveolae of the seta bases completely smooth and shiny. Mesosoma shorter than in *fugax* (ML/CS_480_ 1.155), and less wide (MW/CS_480_ 0.585), always with a moderately deep metanotal groove (MpGr/CS_480_ 0.027). Whole mesosoma smooth and shiny except for 3–6 longitudinal carinulae on lateral metapleuron. Petiole in lateral view with a relatively short peduncle and a high node with a semicircular dorsum, the whole node slightly inclined caudad. Petiole much higher than postpetiole (PEH/CS_480_ 0.368, PPH/CS_480_ 0.292) but in contrast to *fugax* distinctly narrower than postpetiole (PEW/CS_480_ 0.296, PPW/CS_480_ 0.324). Both waist segments completely smooth and shiny. Head, mesosoma, waist, gaster, femora, tibiae, and scape with very abundant, fine and long setae. Pubescence absent. Whole body and appendages light yellowish with a brownish color component which is often more expressed than in *fugax*.

**Figure 20.**
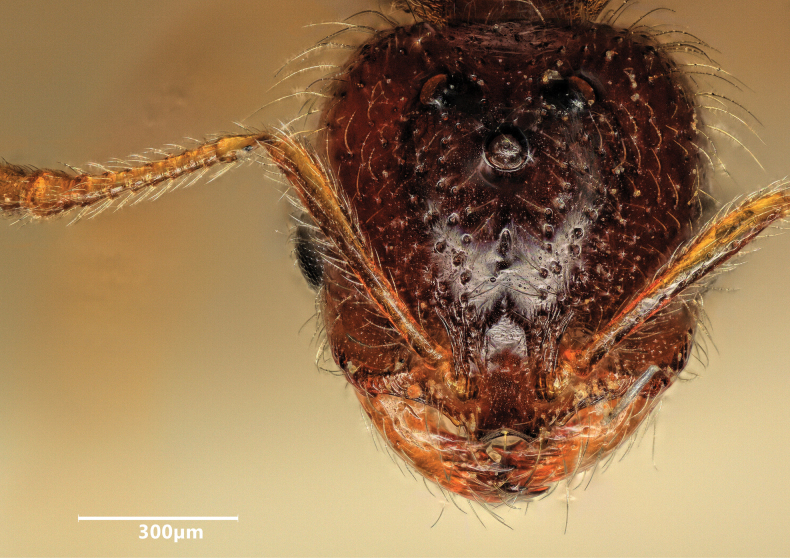
Head of a macrogyne of *Solenopsisjuliae* in full-face view.

**Figure 21.**
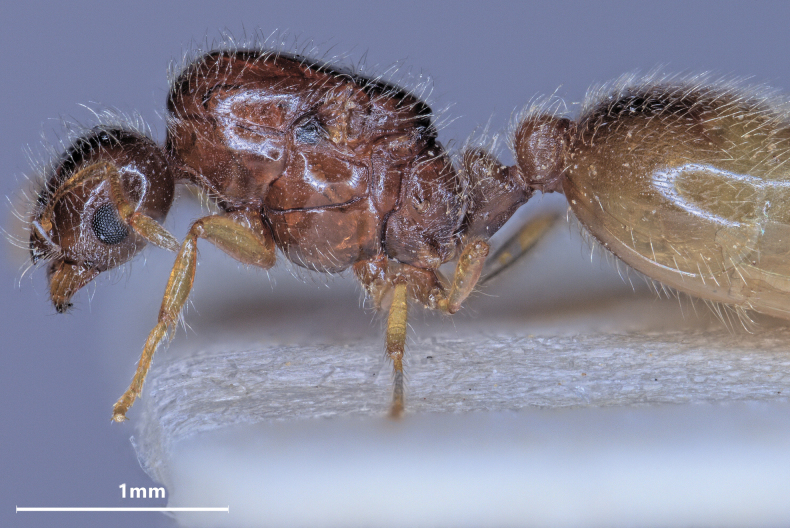
Lateral view of a *Solenopsisjuliae* macrogyne.

***Gyne*** (Table 5, Figs 20, 21): Small, mean CS 798 µm. Head clearly shorter than wide, CL/CW 0.969. Inner third of hind margin of vertex in full face view weakly convex or straight. Scape short, SL/CS 0.657. Frontal carinae short, subparallel and slightly more approached than in *fugax*, FR/CS 0.240. Preocular distance small, PrOc/CS 0.125. Eye moderately large, EL/CS 0.295. Inner clypeal dents spiny and rather long (CLSPLM/CS 0.050), their tips more distant than in *fugax* (CLSPD/CS 0.162) and often slightly diverging. Lateral clypeal dents much less developed than the inner ones (CLSPLL/CS 0.012). Frontal lobes, lateral clypeus and genae carinulate, remaining head surface except for large foveolae of the seta bases completely smooth and shiny, as clearest difference to *fugax* in particular central vertex without any carinulae or rugulae (compare Figs 11, 13, 18). Mesosoma long (ML/CS 1.991) and much higher than wide (MH/CS 1.248, MW/CS 1.016). Whole mesosoma smooth with exception of carinulate lateral scutum and whole metapleuron and anepisternite. Petiole in lateral view with a short peduncle, a high node which is slightly more inclined caudad than in *fugax*; petiole as high as postpetiole (PEH/CS 0.476, PPH/CS 0.470); dorsal crest of petiole node in dorsal view much wider than long, the petiole in contrast to *fugax* much narrower than postpetiole (PEW/CS 0.421, PPW/CS 0.496). Dorsum of both waist segments nearly smooth, the lateral surfaces slightly carinulate. Head, mesosoma, waist, gaster, femora, tibiae, and scape with very abundant, fine and long setae. Pubescence absent. Usually dark to medium brown; legs, antennae and mandibles yellowish.

### ﻿Taxonomic comments

It has been shown that the separation of workers of *S.fugax* and *S.juliae* was very clear in both investigation systems of SC and BS and that gynes were clearly separable with the system of BS. We have also presented an argument that there is most probably no taxon described from Eurasian ranges east of 8°E that could be a senior synonym of *S.juliae*. The exclusion of all 15 taxa described by Bernard (1950, 1959, 1978) from senior synonymy of *S.juliae* is stated here simply for geographic reasons as this species is not known in the western region.

**Figure 22.**
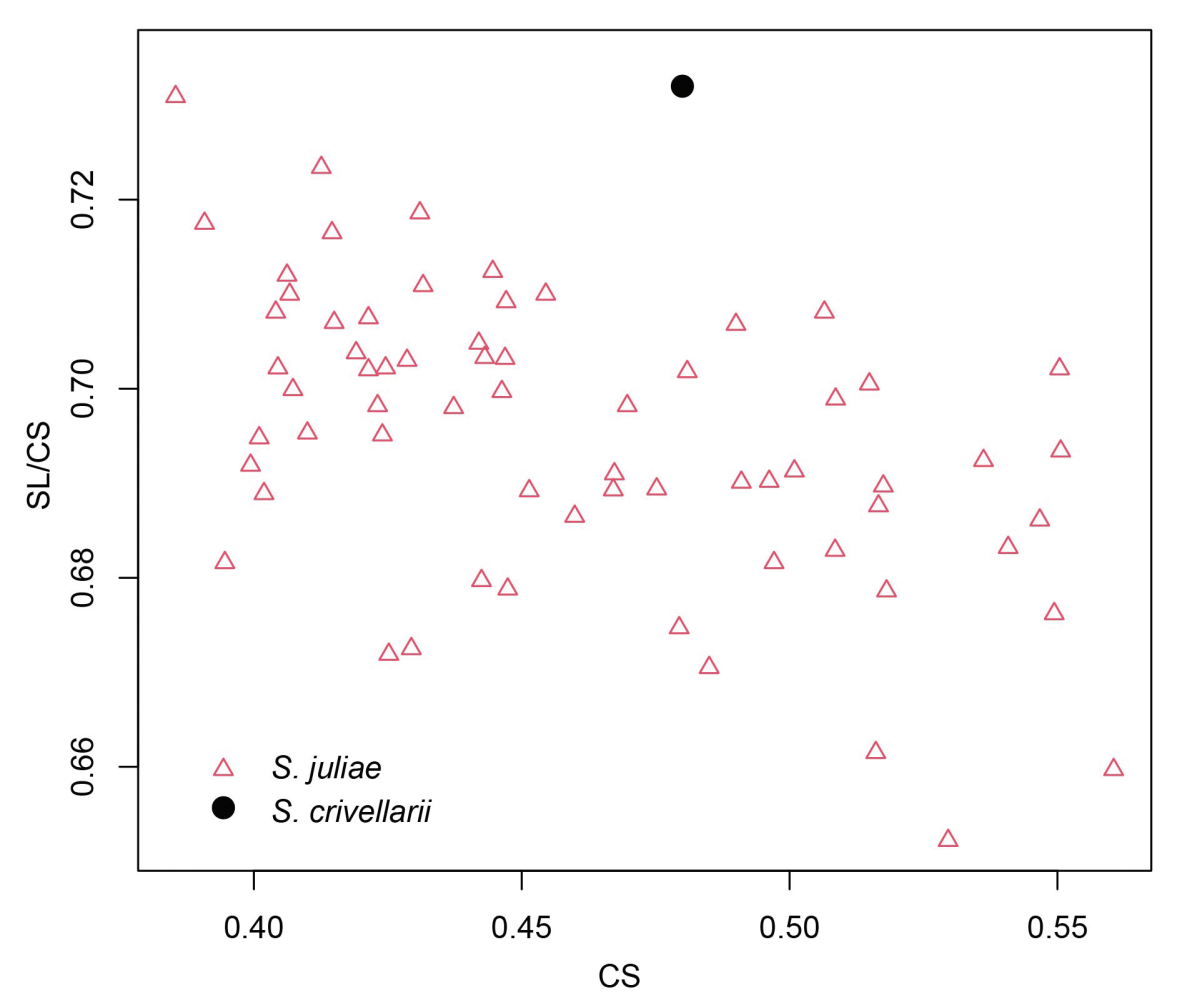
Scatterplot for morphometric ratios of *Solenopsisjuliae* workers and *S.crivellarii* type. Red triangles: *S.juliae*; black dot: *S.crivellarii* type (measured from drawings).

*Solenopsicrivellarii* Menozzi, 1936, described from Diafani (35.76°N, 27.21°E) on the Aegean island of Karpathos, might possibly represent a senior synonym of *S.juliae*. Because the type is not available, and no images are deposited in www.Antweb.org, we studied the original description. The verbal part does not provide any diagnostic characters. The drawings are without scales but concluded from eye size cited as “eyes barely visible in minor workers and only having four or five ommatidia in major workers” (Menozzi 1936: 284, fig. 10) illustrate a major worker. Images of heads imply the risk that scape lengths are depicted as shorter than actual, but a larger length is not suggested (Menozzi 1936: fig. 10). However, the ratio SL/CS derived from the drawing (Menozzi 1936: fig. 10) is 0.732, which is clearly higher than the range known from *S.juliae* (= 0.695 [0.652, 0.730] (*n* = 66)) (see Fig. 22). The frontal lobe distance FL/CS provides an even stronger indication. The depicted specimen (Menozzi 1936: fig. 10) has FL/CS = 0.195, much lower than in the 66 examined *S.juliae* workers of any body size with 0.218 ± 0.007 [0.204, 0.237]. Based on these morphometric arguments and considering the South Aegean insular zoogeography, we conclude that *S.crivellarii* is not a senior synonym of *S.juliae*.

#### ﻿ Solenopsis cypridis

Taxon classificationAnimaliaAsteralesCampanulaceae

Santschi, 1934

1537AF8B-8B0B-5AFE-8EE2-9BC37951133D

Solenopsis fugax var. cypridis Santschi, 1934. [type investigation]

##### Notes.

This species has been described from Limassol in Cyprus.

Four syntype workers and two gynes were investigated from the neotype nest sample, labelled “Chypre 3 Limassol 15. x 30 Mar.....”, “S.fugaxcypridis Sant” and “ANTWEB CASENT0913887”, depository NHM Basel.

##### Diagnosis.

**Figure 23.**
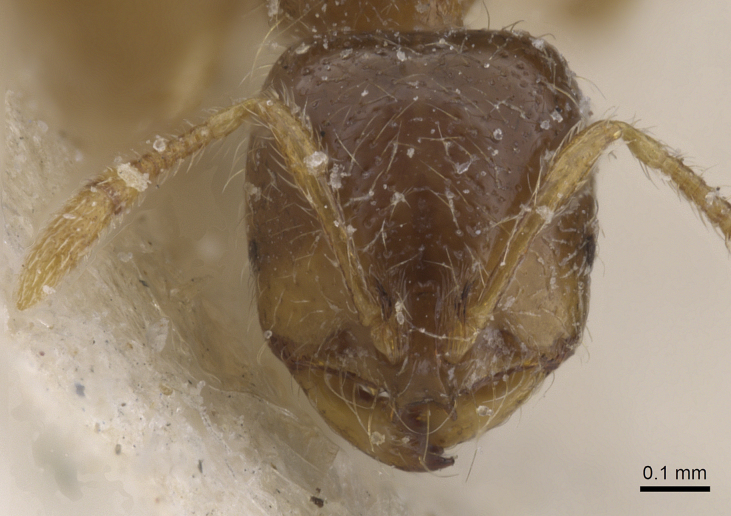
Head of worker of *Solenopsiscypridis* in full-face view (CASENT0913887). Photo: AntWeb.org, Photographer: Will Ericson.

**Figure 24.**
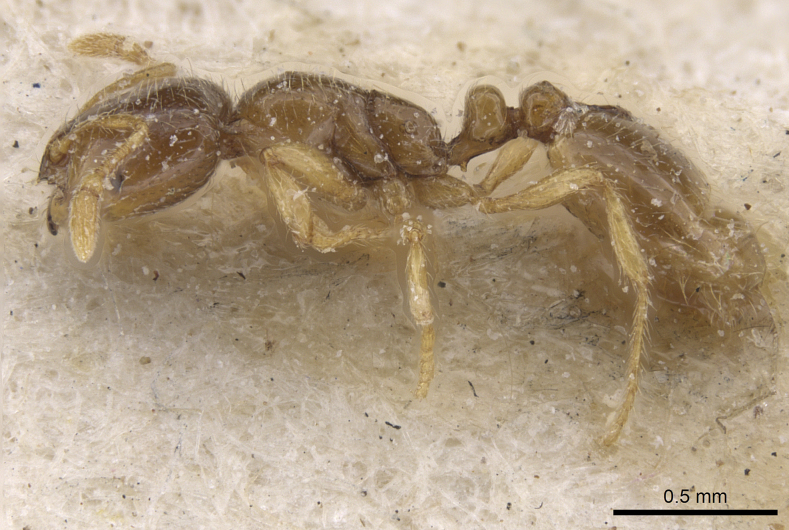
Lateral view of *Solenopsiscypridis* worker (CASENT0913887). Photo: AntWeb.org, Photographer: Will Ericson.

***Worker*** (Table 4; Figs 23, 24; images CASENT0913887 in www.antweb.org): larger than *fugax*, mean CS = 515 µm. All shape ratios given below are mean values allometrically corrected for CS = 480 µm. Head less elongated than in *fugax*, CL/CW_480_ 1.159. Hind margin of vertex in full face view straight. Scape short, SL/CS_480_ 0.696. Frontal carinae short, parallel, FL/CS_480_ 0.230, FR/CS_480_ 0.230. Preocular distance rather large, PrOc/CS_480_ 0.200. Eye small, EL/CS_480_ 0.095. Inner clypeal dents spiny and moderately long (CLSPLM/CS_480_ 0.067), their tips diverging and as result more distant than in *fugax* (CLSPD/CS_480_ 0.154). Lateral clypeal dents much less developed (CLSPLL/CS_480_ 0.021). Frontal lobes carinulate, whole surface vertex except for numerous foveolae of the seta bases completely smooth and shiny. Mesosoma shorter than in *fugax* (ML/CS_480_ 1.186) but similarly wide (MW/CS_480_ 0.600), always with a moderately deep metanotal groove (MpGr/CS_480_ 0.035). Whole mesosoma smooth and shiny except for 3–6 longitudinal carinulae on lateral metapleuron. Petiole in lateral view with a short peduncle and a high node the dorsum of which is broader than in *fugax*; the whole node slightly inclined caudad. Petiole much higher and only slightly narrower than postpetiole (PEH/CS_480_ 0.375, PPH/CS_480_ 0.307, PEW/CS_480_ 0.311, PPW/CS_480_ 0.322). Both waist segments completely smooth and shiny. Head, mesosoma, waist, gaster, femora, tibiae, and scape with very abundant, fine and long setae. Pubescence absent. Head, mesosoma, waist, and gaster dirty brown (i.e., darker than in *fugax*); appendages contrastingly light yellowish.

***Gyne*** (Table 5): Larger than *fugax*, mean CS = 972 µm. Head shorter than in *fugax*, CL/CW 0.890, a smaller head length index proves true also after removal of allometric variance. Scape short, SL/CS 0.691. Frontal carinae short, subparallel and much more distant than in *fugax*, FR/CS 0.281, a wider frons also proves true also after removal of allometric variance. Preocular distance small, PrOc/CS 0.126. Eye slightly larger than in *fugax*, EL/CS 0.304; this difference is increased after removal of allometric variance. Inner clypeal dents spiny and rather long (CLSPLM/CS 0.055), their tips much more distant than in *fugax* (CLSPD/CS 0.174). Lateral clypeal dents much less developed than the inner ones (CLSPLL/CS 0.014). Mesosoma long (ML/CS 2.094) and much higher than wide (MH/CS 1.334, MW/CS 1.154).

### ﻿Differential diagnosis and rank elevation

Based on the following arguments, we consider *Solenopsiscypridis* as a species separate from *S.fugax* – either valid or representing a junior synonym of another East Mediterranean taxon. Since *Solenopsiscypridis* is not a synonym of *S.fugax* or *S.juliae*, its taxonomic status is beyond the scope of this paper and must be clarified in the future. The gynes differ from *fugax* by larger absolute sizes and larger FR/CS and CLSPD/CS. The differences in the latter indices prove true with removal of allometric variance (Table 5). A principal component analysis considering the characters CS, SL/CS, EL/CS, FL/CS, FR/CS, and CLSPD/CS separates the type gynes from all gynes of *fugax* and *juliae* (Fig. 7). This separation is repeated by the PCA in the workers using RAV-corrected data of CL/CW_480_, CLSPD/CS_480_, CLSPLM/CS_480_, SL/CS_480_, FR/CS_480_, PEW/CS_480_, PEH/CS_480_ and ML/CS_480_ (Fig. 6).

## Supplementary Material

XML Treatment for Solenopsis fugax

XML Treatment for Solenopsis flavidula

XML Treatment for Solenopsis (Diplorhoptrum) fugax var. pontica

XML Treatment for Solenopsis (Diplorhoptrum) fugax var. scytica

XML Treatment for Solenopsis (Diplorhoptrum) fugax var. furtiva

XML Treatment for Solenopsis juliae

XML Treatment for Solenopsis nitida

XML Treatment for Solenopsis cypridis

## ﻿Additional information

### Author ORCIDs

Sándor Csősz https://orcid.org/0000-0002-5422-5120

Bernhard Seifert https://orcid.org/0000-0003-3850-8048

Zalimkhan M. Yusupov https://orcid.org/0000-0002-5149-9679

Gábor Herczeg https://orcid.org/0000-0003-0441-342X

### Data availability

All of the data that support the findings of this study are available in the main text or Supplementary Information.

## Supplementary materials

Supplementary material 1 List of morphometrically investigated samples Data type xls Explanation note Unique codes for nest samples, specimens investigated by BS and SC, locality, geographic coordinates (Latitude, Longitude) in decimal format, altitude (Elevation) in meters a.s.l., collector’s name, date and depositories are provided. Unique CASENT numbers are given for type specimens. https://binary.pensoft.net/file/956129This dataset is made available under the Open Database License (http://opendatacommons.org/licenses/odbl/1.0/). The Open Database License (ODbL) is a license agreement intended to allow users to freely share, modify, and use this Dataset while maintaining this same freedom for others, provided that the original source and author(s) are credited.Sándor Csősz, Bernhard Seifert, Márk László, Zalimkhan M. Yusupov, Gábor Herczeg

Supplementary material 2 Morphometric data of 15 continuous morphometric traits of 203 individuals collected by SC is given in µm Data type xlsx https://binary.pensoft.net/file/956130This dataset is made available under the Open Database License (http://opendatacommons.org/licenses/odbl/1.0/). The Open Database License (ODbL) is a license agreement intended to allow users to freely share, modify, and use this Dataset while maintaining this same freedom for others, provided that the original source and author(s) are credited.Sándor Csősz, Bernhard Seifert, Márk László, Zalimkhan M. Yusupov, Gábor Herczeg

Supplementary material 3 Morphometric data of 18 continuous morphometric traits of 171 individuals collected by BS is given in mm Data type xlsx https://binary.pensoft.net/file/956131This dataset is made available under the Open Database License (http://opendatacommons.org/licenses/odbl/1.0/). The Open Database License (ODbL) is a license agreement intended to allow users to freely share, modify, and use this Dataset while maintaining this same freedom for others, provided that the original source and author(s) are credited.Sándor Csősz, Bernhard Seifert, Márk László, Zalimkhan M. Yusupov, Gábor Herczeg

Supplementary material 4 R script of NC clustering and method PART implementing cluster methods “hclust” and “kmeans”, including “Mark dendrogram” function mapping the results of partitioning algorithm PART on the dendrogram Data type txt https://binary.pensoft.net/file/956132This dataset is made available under the Open Database License (http://opendatacommons.org/licenses/odbl/1.0/). The Open Database License (ODbL) is a license agreement intended to allow users to freely share, modify, and use this Dataset while maintaining this same freedom for others, provided that the original source and author(s) are credited.Sándor Csősz, Bernhard Seifert, Márk László, Zalimkhan M. Yusupov, Gábor Herczeg
